# Wood Vault: remove atmospheric CO_2_ with trees, store wood for carbon sequestration for now and as biomass, bioenergy and carbon reserve for the future

**DOI:** 10.1186/s13021-022-00202-0

**Published:** 2022-04-01

**Authors:** Ning Zeng, Henry Hausmann

**Affiliations:** 1 Department of Atmospheric and Oceanic Science, University of Maryland, College Park, USA; 2 Earth System Science Interdisciplinary Center, University of Maryland, College Park, USA; 3 Department of Geology, University of Maryland, College Park, USA; 4 Maryland Energy Innovation Institute, University of Maryland, College Park, USA

## Abstract

**Background:**

Wood harvesting and storage (WHS) is a hybrid Nature-Engineering combination method to combat climate change by harvesting wood sustainably and storing it semi-permanently for carbon sequestration. To date, the technology has only been purposefully tested in small-scale demonstration projects. This study aims to develop a concrete way to carry out WHS at large-scale.

**Results:**

We describe a method of constructing a wood storage facility, named Wood Vault, that can bury woody biomass on a mega-tonne scale in specially engineered enclosures to ensure anaerobic environments, thus preventing wood decay. The buried wood enters a quasi-geological reservoir that is expected to stay intact semi-permanently. Storing wood in many environments is possible, leading to seven versions of Wood Vault: (1) Burial Mound (Tumulus or Barrow), (2) Underground (Pit, Quarry, or Mine), (3) Super Vault, (4) Shelter, (5) AquaOpen or AquaVault with wood submerged under water, (6) DesertOpen or DesertVault in dry regions, (7) FreezeVault in cold regions such as Antarctica. Smaller sizes are also possible, named Baby Vault. A prototype Wood Vault Unit (WVU) occupies 1 hectare (ha, 100 m by 100 m) of surface land, 20 m tall, stores up to 100,000 m^3^ of wood, sequestering 0.1 MtCO_2_. A 1 MtCO_2_ y^−1^ sequestration rate can be achieved by collecting currently unused wood residuals (WR) on an area of 25,000 km^2^, the size of 10 typical counties in the eastern US, corresponding to an average transportation distance of less than 100 km. After 30 years of operation, such a Wood Vault facility would have sequestered 30 MtCO_2_, stored in 300 WVUs, occupying a land surface of 300 ha. The cost is estimated at $10–50/tCO_2_ with a mid-point price of $30/tCO_2_. To sequester 1 GtCO_2_ y^−1^, wood can be sourced from currently unexploited wood residuals on an area of 9 Mkm^2^ forested land (9 million square kilometers, size of the US), corresponding to a low areal harvesting intensity of 1.1 tCO_2_ ha^−1^ y^−1^. Alternatively, giga-tonne scale carbon removal can be achieved by harvesting wood at a medium harvesting intensity of 4 tCO_2_ ha^−1^ y^−1^ on 3 Mkm^2^ of forest (equivalent to increasing current world wood harvest rate by 25%), or harvest on 0.8 Mkm^2^ forest restored from past Amazon deforestation at high harvest intensity, or many combinations of these and other possibilities. It takes 1000 facilities as discussed above to store 1 GtCO_2_ y^−1^, compared to more than 6000 landfills currently in operation in the US. After full closure of a Wood Vault, the land can be utilized for recreation, agriculture, solar farm, or agrivoltaics. A more distributed small operator model (Baby Vault) has somewhat different operation and economic constraints. A 10 giga-tonne sequestration rate siphons off only 5% of total terrestrial net primary production, thus possible with WHS, but extreme caution needs to be taken to ensure sustainable wood sourcing.

**Conclusions:**

Our technical and economic analysis shows that Wood Vault can be a powerful tool to sequester carbon reliably, using a variety of wood sources. Most pieces of the technology already exist, but they need to be put together efficiently in practice. Some uncertainties need to be addressed, including how durability of buried wood depends on detailed storage methods and burial environment, but the science and technology are known well enough to believe the practicality of the method. The high durability, verifiability and low-cost makes it already an attractive option in the current global carbon market. Woody biomass stored in Wood Vaults is not only a carbon sink to combat current climate crisis, but also a valuable resource for the future that can be used as biomass/bioenergy and carbon supply. The quantity of this wood utilization can be controlled carefully to maintain a desired amount of CO_2_ in the atmosphere to keep the Earth’s climate from diving into the next ice age, acting as a climate thermostat. The CO_2_ drawdown time is on the order of 100 years while the ramp-up time is a decade. A sense of urgency is warranted because the CO_2_ removal rate is limited by biosphere productivity, thus delayed action means a loss of opportunity. In conclusion, WHS provides a tool for managing our Earth system, which will likely remain forever in the Anthropocene.

## Background

To achieve the goals of the Paris climate accord and the Glasgow climate pact of keeping global mean temperature increases below 1.5–2 °C, carbon sequestration via negative emissions technology (NET) will be needed to augment emissions reduction [1]. Additionally, it is not enough to simply remove CO_2_ from the atmosphere, but also necessary to sequester it reliably for hundreds of years or longer [2].

Wood harvesting and storage (WHS) has been proposed as one such method [3, 4]. In this method, sustainably harvested or collected woody biomass are buried underground in anaerobic condition or stored in other environments designed to prevent decomposition, forming an effective carbon sink.

Compared to many nature-based methods, WHS is a hybrid Nature-Engineering approach, with the key difference of being continual (no-saturation) and semi-permanent storage enabled by engineering methods. On the WHS method, a recent US National Academy of Sciences (NAS) report [2] states that “[WHS]…could be viable approaches to increasing carbon removal… To date, this proposed approach has not been tested though the technology is simple and easily applied”. Actually, a limited number of projects have been conducted [5], but are not at the scale or quality needed. Financial and societal challenges remain before the method can be applied at large scale.

## Methods

**Fig. 1:**
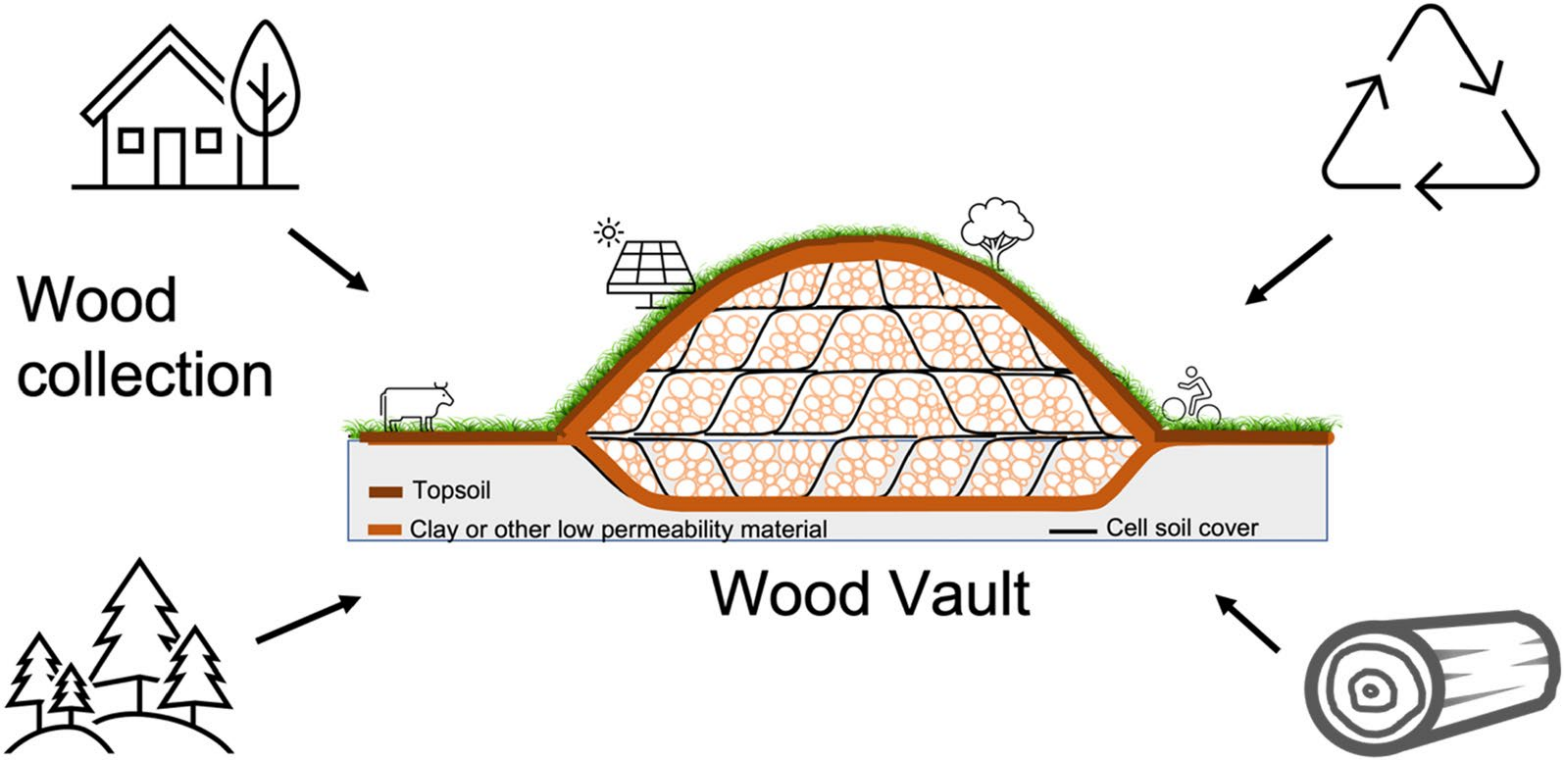
Wood Vault is a centralized storage facility that collects wood from a variety of sources such as urban natural wood residuals (woody yard trimmings, NWR) , wood from storm damage, wood from forest thinning, construction and demolition debris, wood harvested in sustainably managed forests. The buried biomass is sealed off from oxygen with clay or other low-permeability material and embedded in a subterranean environment that will prevent decomposition semi-permanently. Variations and other versions are discussed later

Here we propose a concrete way to carry out WHS. The method envisions centralized facilities to store wood semi-permanently. We name such a facility Wood Vault because it stores wood in a specially engineered enclosure that protects it from decay, with the potential for future use. It is valuable now as sequestered carbon to mitigate climate change, and in the future as a reserve of carbon, biomass and bioenergy. Such large facilities are particularly suitable for collecting and storing wood acquired from diffused sources. Three key aspects of the vision are (Fig. 1):

1. Collect and stockpile wood from surrounding regions, such as natural wood residuals (NWR) or sustainably harvested wood.
2. Construct the facility by digging a trench, filling it with wood, and enclosing the wood in a way that maintains an anaerobic condition.
3. After enclosure of the Wood Vault, use the land for recreation, agriculture, or solar farming.

In the results below, we describe the technical details to carry out these steps. Several versions of Wood Vault are proposed to create anaerobic, dry, or cold environments, conditions ideal for wood preservation. Data from literature are also used to estimate the carbon sequestration rate and economics of operating a Wood Vault facility. Data used are described in the text and the Appendix.

## Results

### Woody biomass availability and wood sourcing

#### General types of wood sourcing and their potential for CO_2_ removal

**Fig. 2:**
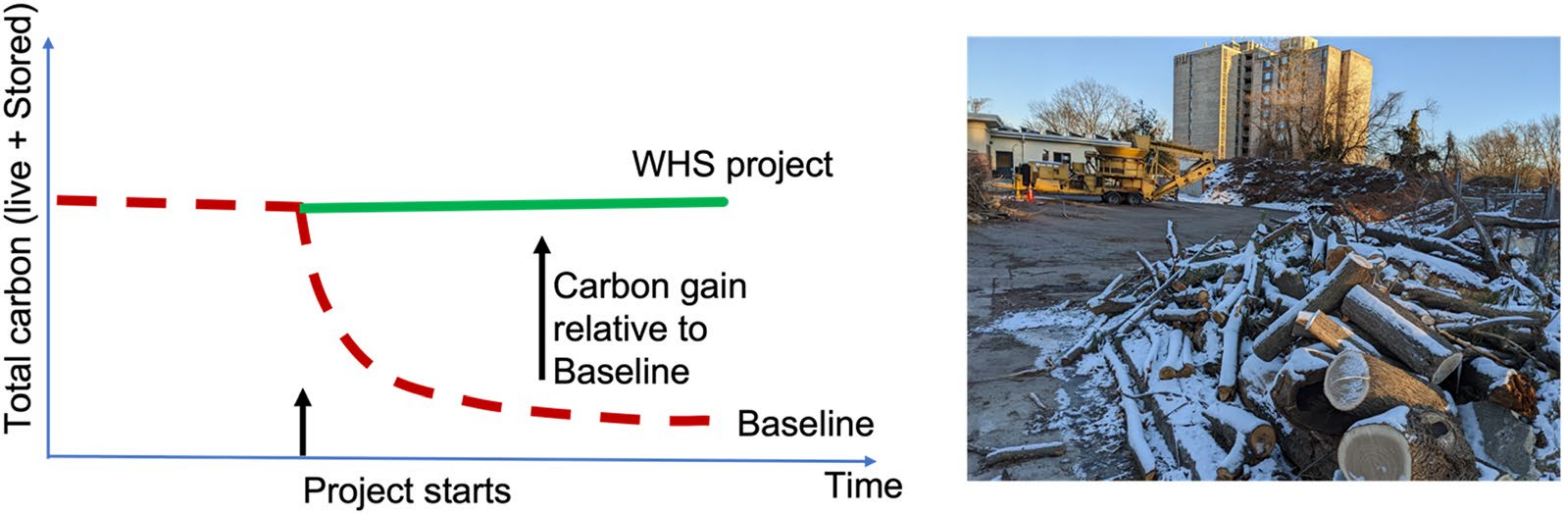
Type-A wood sourcing for WHS: Opportunistic sources such as urban natural wood residuals (NWR) that would decompose within a short amount of time (Baseline scenario, red-dashed line), whereas a WHS project (Project scenario, green line) stores it semi-permanently. The difference between the two scenarios (Project minus Baseline) is the carbon gain that serves as the basis for carbon accounting. Photo: Urban wood collected at the municipal public works facility, City of Takoma Park, Maryland. Photo by Ning Zeng

**Fig. 3:**
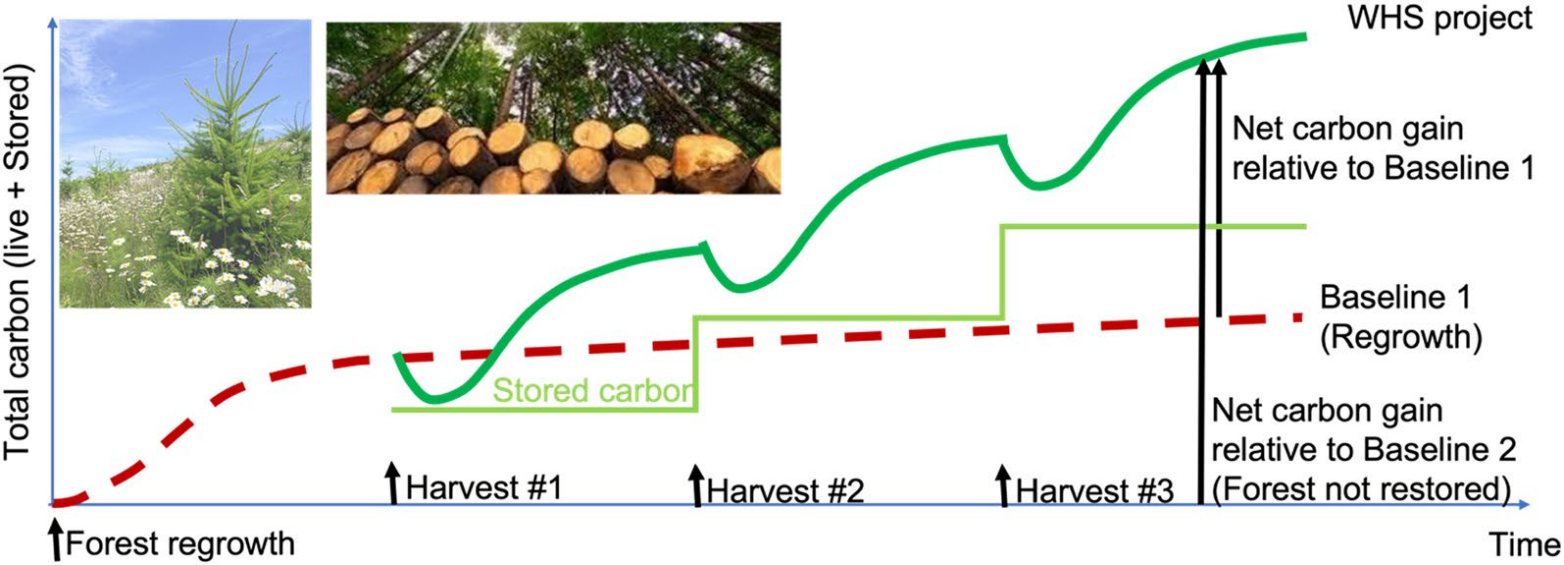
Type-B wood sourcing from a sustainably managed secondary-growth forest that grows back from disturbance such as fire, storm damage, deforestation. The grow-back can be either natural or facilitated (planted). The total carbon on land (green line) consists of the active forest (live vegetation, litterfall and soil carbon) plus the stored carbon from the WHS Project (lime green). The net carbon gain of the Project (green line) can be counted relative to regrowth without WHS (Baseline 1, red-dashed line, starting from Harvest #1), or no-regrowth (Baseline 2, red-dashed line, starting from forest regrowth at time = 0). Photo insets: forest regrowth after disturbance and harvested wood ( source: Wikipedia)

We consider two types of wood sources: Type-A or opportunistic sources (Fig. 2) and Type-B or harvested wood from sustainably managed forests (Fig. 3).

In Type-A wood sourcing, woody biomass is collected from opportunistic sources, often with environmental co-benefits such as fire risk reduction and waste utilization. They are ‘low-hanging fruits’, limited by the availability of ‘waste’ wood. They are often one-time opportunities such as the utilization of urban ‘waste wood’. This method may have a potential of up to 1 GtCO_2_ per year globally [4].

Opportunistic sources are not reliable individually. However, over a large enough area, there will be a near steady supply for a large facility, with the amount fundamentally controlled by forest productivity in the region, except for regions with large wood imports/exports. Opportunistic sources include urban wood residuals (WR, wood trimmings from backyard tree removal, construction and demolition debris, old furniture and wood pallets etc.), land clearing for development and agriculture, logging residue, thinning for precommercial treatment, thinning for fuel treatment to reduce fire risk, and wood processing residue. In the US, it is estimated that a total of 328 Mt (mega-tonnes) of wet biomass is unexploited annually [6], corresponding to a 1.1 tCO_2_ ha^−1^ y^−1^ availability if distributed over 3 Mkm^2^ (3 million square kilometers, US forested area) (see details in Appendix). Throughout this paper, we approximate 1 tonne of wet/green biomass as 1 tonne of CO_2_ sequestered.^1^ For estimating waste wood collection area for a large storage facility below, we will use a benchmark value of 0.4 tCO_2_ ha^−1^ y^−1^, which we consider immediately available at no or low cost to the facility as it is simply to make a ‘waste’ valuable.

For Type-B sourcing, wood is harvested from forests managed for carbon sequestration (fully or partially). Here we consider two benchmark values: a medium harvesting intensity of 4 tCO_2_ ha^−1^ y^−1^, and a high harvesting intensity of 12 tCO_2_ ha^−1^ y^−1^, based on the top-down estimates of Zeng et al. [4] using forest coarse wood productivity. If conducted at the medium harvesting intensity over an area of 3 Mkm^2^ (current US forested area), it will lead to 1 GtCO_2_ y^−1^ sequestration (Table 1), compared to 0.5–3 GtCO_2_ y^−1^ needed to offset hard-to-cut fossil fuel emissions in a renewable energy dominated future [7]. At the high intensity of 12 tCO_2_ ha^−1^ y^−1^ over an area of 8 Mkm^2^ (about the size of the US, or 20% of the world’s total forested area), we can sequester 10 GtCO_2_ y^−1^, the total sequestration needed from NETs (assumed to be all BECCS in the IPCC 1.5 °C scenario) [1]. The 10 GtCO_2_ y^−1^ rate corresponds to the high end of the potential of 3–10 GtCO_2_ y^−1^ estimated using a top-down approach with world total forest productivity constrained by land use, topography, conservation and existing wood use [4]. In particular, even this high-end estimate assumed that 50% tropical forests and 20% midlatitude forests are kept intact. While the high 10 GtCO_2_ y^−1^ rate is indeed possible and can be implemented in a climate emergency, it should be carried out with great caution. On the other hand, the lower values on few giga-tonne scale can be achieved quite sustainably.

**Table 1:** Targets of carbon sequestration amount and example scenarios to achieve them using WHS

Target rate of carbon sequestration	Examples to achieve the goal without expansion of current wood harvest (use only unexploited wood residuals)		Examples with expansion or repurposing of forest management and harvest	
			Medium harvest intensity (4 tCO_2_ ha^−1^ y^−1^)	High harvest intensity (12 tCO_2_ ha^−1^ y^−1^); fast growing species
1 MtCO_2_ y^−1^	Unused urban wood residue on 25,000 km^2^ (size of MD, or 1/6 of NC) at 0.4 tCO_2_ ha^−1^	Forest thinning for fuel treatment on 25,000 km^2^	On 2500 km^2^ of forested land (size of two counties in eastern US)	On 800 km^2^ (30 km by 30 km forest, land area of New York City)
1 GtCO_2_ y^−1^	Most unexploited wood residue from 9 Mkm^2^ (temperate forested land the size of US; US wood utilization rate) at 1.1 tCO_2_ ha^−1^ intensity	25% of current world wood harvest rate	2.5 Mkm^2^ of forest	0.8 Mkm^2^ restored Amazon rainforest (area deforested since 1970)
10 GtCO_2_ y^−1^ (27% of 2020 fossil fuel emissions; total NETs needed in IPCC 1.5 °C scenario)			25 Mkm^2^ forest land (about half of total world forest)	8 Mkm^2^ of productive forest land (slightly less than the size of US/China)

MD: the state of Maryland; NC: the state of North Carolina. Mkm^2^: million square kilometers, or 100 million hectares. A targeted rate can be fulfilled with a specific wood sourcing option listed, but in practice more likely by a combination of multiple choices because the best option depends on the local circumstances

To give a few examples, to sequester 1 GtCO_2_ y^−1^, a level achievable without dramatic transformation of the industry, wood can be sourced from currently unexploited wood residue on an area of 9 Mkm^2^ (9 million square kilometers, size of the US/China) (low intensity), or by increasing current world wood harvest rate by 25% (equivalent to harvesting wood at a medium harvest intensity of 4 tCO_2_ ha^−1^ y^−1^ on 3 Mkm^2^ of forest), or harvest on 0.8 Mkm^2^ of restored Amazon forest at high harvest intensity of 12 tCO_2_ ha^−1^ y^−1^, or a combination of multiple possibilities.

#### Size of wood collection area for a single large facility

Here we only consider wood residuals collected from urban-suburban regions, for example, an area of 2500 km^2^ (50 km by 50 km), the size of two typical counties in the eastern United States.

The wood availability of 0.4 tonne ha^−1^ y^−1^ over 2500 km^2^ yields 100,000 tonne y^−1^. After 10 years, a total of 1 Mt will be collected. For rural area with harvesting from managed forest, at 10 times higher wood availability, or a larger area of 25,000 km^2^, the first year will already collect 1 Mt. Intermediate scenarios are also possible by collecting wood from somewhat larger area (more than a couple of counties for suburban, at added transportation expense), or a combination of these. A summary is given in Table 2.

**Table 2:** Wood availability per unit area for large-scale storage facility depending on the method of wood sourcing, based on US forestry data (see Appendix)

Method	Potential wood availability (tCO_2_ ha^−1^ y^−1^)	Size of wood collection area for large storage facility	Assumptions in the estimated potential
Urban-suburban wood residuals	0.4	2500 km^2^	Utilize 30–50% of unexploited urban wood residuals
Rural (Managed forest)	4	2500 km^2^	Utilize most of wood residuals and forestry residue, or sustainably managed forest
Combination	0.4–4	2500–25,000 km^2^	Collection over a larger area or high rate

A Wood Vault facility can have multiple WVUs that are built up over time. We suggest one facility for an area the size of a few counties in the US, constraint by the availability and sustainability of wood sourcing.

#### Project-level prototypes of wood sourcing

**Fig. 4:**
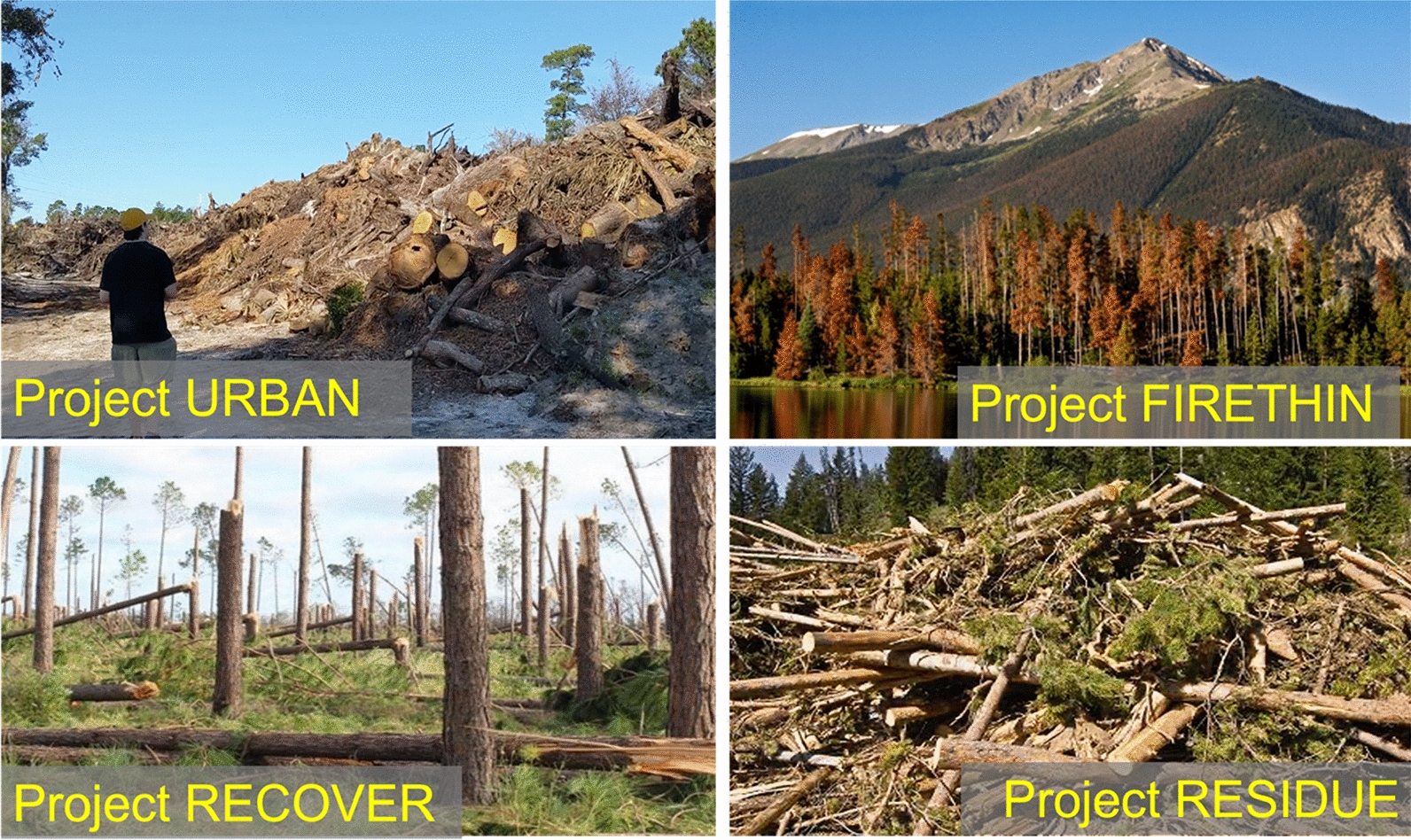
Type-A opportunities for sourcing wood for WHS, with co-benefits: (1) Project URBAN: utilize currently unexploited urban wood residuals; (2) Project FIRETHIN: utilize woody biomass from forest thinning for fire risk reduction; (3) Project RECOVER: utilize storm-damaged woody biomass by hurricanes and tornados; (4) Project RESIDUE: forestry residue from timber harvest and other operations

While the ‘top-down’ assessment above provides a panoramic view of wood sourcing potential, here we list several ‘bottom-up’ project-level opportunities. Firstly, under Type-A (opportunistic sources) we propose (Fig. 4):

1. Project URBAN: utilize currently unexploited urban wood residuals. Biomass from urban tree removal, construction site tree removal, demolition and old furniture wood. These are often a burden and may be costly to dispose. Collect and bury this biomass for carbon sequestration can completely reverse the cost equation.
2. Project FIRETHIN: Woody biomass from forest thinning for fire risk reduction, precommercial treatment and other purposes is used for sequestration. Fire suppression, such as in the America West over the last century, has left a large amount of dead vegetation on the forest floor. Combined with drought and insect infestation, this has led to more frequent and larger fires in recent years. Additionally, the release of this carbon pool through catastrophic fires may become an important source of atmospheric CO_2_ in the future. Collecting dead trees or thinning and burying them would reduce fire danger while creating an effective carbon sink (relative to letting it burn or rot).
3. Project RECOVER: utilize naturally damaged woody biomass caused by natural disasters such as hurricanes, tornados, fire, aging/death, and disease. Recovering trees from storm blowdown and other natural or unnatural disasters and storing the carbon will prevent the release of this carbon into the atmosphere. It’s a loss of opportunity if we don’t do it.
4. Project RESIDUE: utilize forestry residue from timber harvests and other operations. Residues from forestry operations include slash and woodchips. In many places, they are not utilized due to economic and other constraints. To the degree that nutrient and other ecosystem functions are sufficiently maintained, a portion can be buried for carbon sequestration. Furthermore, careful management enabled by the carbon value has the potential to support better ecosystem functioning. Compared to whole wood logs, the smaller-size woody biomass such as woodchips may not preserve as well, so their permanence after burial needs to be better established before large-scale implementation.

**Fig. 5:**
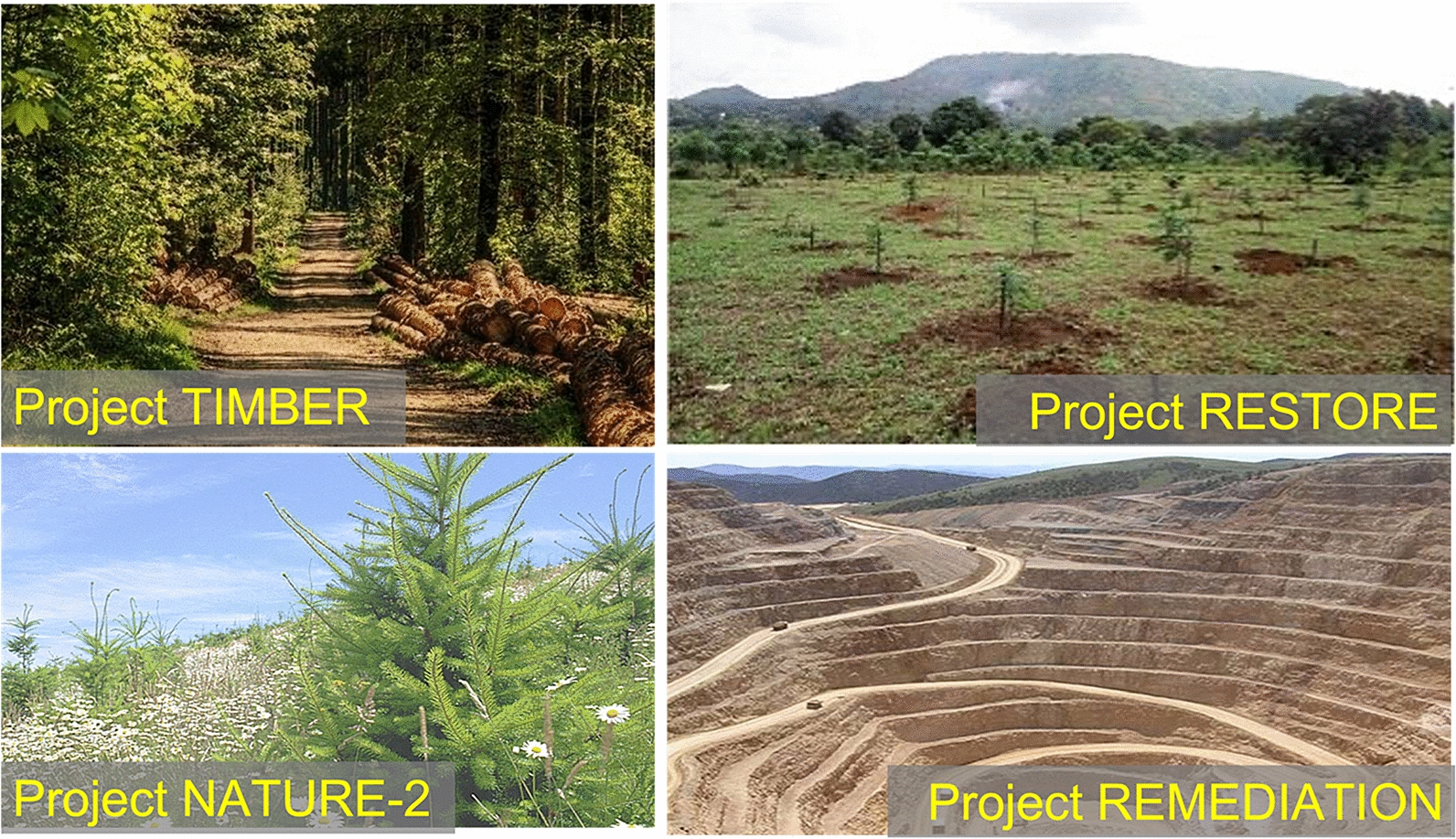
Prototype projects with Type-B wood sourcing: (1) Project TIMBER: utilize partially or fully the wood harvested from existing timberland; (2) Project RESTORE: utilize wood from forest restored from degraded or marginal land; (3) Project Nature-2: utilize wood from forest recovered from natural disturbance/death. Type-B projects, when implemented globally, have the potential for multi-gigatonne scale carbon sequestration. Also shown is Project REMEDIATION, not in terms of wood sourcing, but using abandoned quarry/mine as burial site with co-benefits of carbon sequestration and mine remediation

Type-B projects, with wood sourcing by harvesting different types of forest, when implemented globally, have the potential for multi-gigatonne scale annual carbon sequestration (Fig. 5).

1. Project TIMBER: Wood is harvested from a managed forest such as timberland. The forest is often privately owned, and has been used for timber, pulp for paper, biomass or bioenergy for many years. The sustainability and environmental impact of the forest are generally well established. Wood can be sustainably removed via thinning, rotation or other schemes. Carbon sequestration re-purposing adds a new revenue to the original forest management objectives. Other traditional uses of wood such as furniture should have priority, but there is more wood available beyond the traditional market demand.
2. Project RESTORE: utilize wood from forests restored from degraded or marginal land. Carrying out WHS after initial forest establishment will extend indefinitely the carbon sequestration potential of reforestation/afforestation projects whose carbon sink approaches saturation after some decades. This synergistic carbon benefit that accrues over time is seen clearly in Fig. 3. This fundamentally changes the equation of the climate benefit of reforestation, acting as a strong incentive for supporting reforestation/afforestation as soon as possible. Because these restored forests are, by definition, managed, so there is no issue with conservation. Of course, sustainability should be included as part of the WHS project methodology requirement for carbon credits. For example, tropical deforestation leaves land in poor quality after some years of grazing and agriculture. As an idealized scenario, a sequestration rate of 1 GtCO_2_ y^−1^ can be achieved if the 0.8 Mkm^2^ restored Amazon rainforest (area deforested since 1970) is fully utilized with WHS at high harvest intensity (Table 1), though in practice we recommend mixed use.
3. Project NATURE-2: utilize wood from forests recovered from natural disturbance/death (secondary regrowth forest). Wood is harvested from a secondary-growth forest, that is, a forest growing back from agricultural abandonment, degradation, fire or other disturbances. Such a forest goes through initial growth, followed by self-thinning, natural death, disease and other processes. Active management can lead to an overall more productive forest and better ecosystem service, while producing a carbon sequestration stream.
4. At this point of time, we warn against using old-growth and other conservation forests for WHS.

Also shown in Fig. 5 is Project REMEDIATION, not in terms of wood sourcing, but using an abandoned quarry/mine as a burial site with the co-benefits of carbon sequestration and mine remediation. In this case, wood needs to be sourced with one or more of the above wood sourcing methods.

In the analysis below, we use urban wood residuals as an example (Project URBAN) because this is an immediately available ‘low-hanging fruit’, while recognizing that some aspects may differ for other types of wood sourcing. In general, for Type-A wood sourcing, no major policy incentives are needed other than allowing the stored carbon to be eligible in current carbon markets such as EUETS at the recent €50–100/tCO_2_, the US IRS 45Q tax credit at $35–50/tCO_2_, and many other national and regional carbon markets [8], or the burgeoning voluntary markets [8, 9]. It is straightforward to extrapolate our estimates to higher wood availability on managed forests which is also quite feasible in rural regions such as the US Southeast. If we include the medium harvest intensity 4 tCO_2_ ha^−1^ y^−1^ on managed forest, the facility can be either larger to collect wood from a similar area, or similar size with wood from a smaller area.

### Wood Vault as carbon storage facility

#### Wood Vault: the concept

**Fig. 6:**
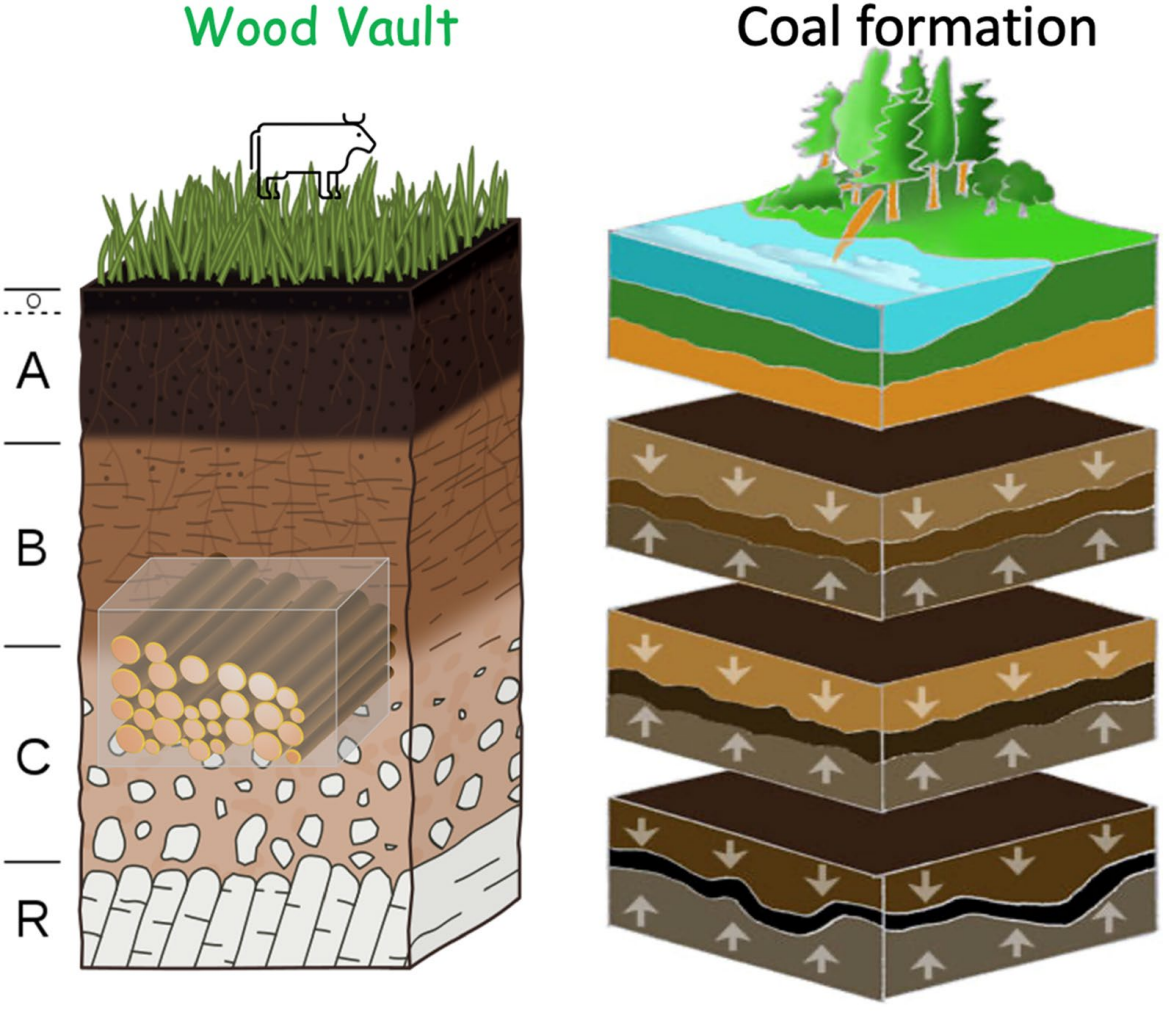
The essence of Wood Vault in relation to a standard soil profile: Wood is buried in the subsoil (B-horizon) or lower horizon, completely isolated from the biologically active topsoil above. The wood is capped with clay or other low-permeability material to ensure anaerobic condition. If the original subsoil already has sufficiently low permeability, no other material needs to be imported. After enclosure, the top is backfilled with original topsoil and organic layer to allow shallow vegetation cover or other use. The semi-transparent cubic enclosure is only conceptual as the actual material and engineering methods are described in the various versions of Wood Vault. Source of background graphics: Wikipedia

We propose the term Wood Vault to describe a class of specially engineered structures that keep woody biomass from decomposing or being otherwise damaged, for the purpose of semi-permanent wood storage as carbon sequestration and biomass/bioenergy reserves. The main type of Wood Vault uses soil. Other types of Wood Vaults in water and dry or cold conditions are also possible. The illustration in Fig. 6 shows the essence of the soil category of Wood Vault in relation to a standard soil profile. The key factors of a Wood Vault are:

- Woody biomass, raw or minimally processed, is buried deep underground, isolated from the biologically active topsoil above.
- The wood is buried in a way to ensure anaerobic condition or stored in dry or cold conditions in order to prevent decomposition, leading to durable semi-permanent storage.
- The WHS process is akin to the first step of coal formation. Compared to natural coal formation in which plants are buried in fortuitous conditions at slow geological rate, the rate of WHS-style wood burial is accelerated by human intervention via wood harvesting and Wood Vault construction. This is the exact opposite of accelerated oxidation of fossil fuel by dig-and-burn. WHS is a ‘near-perfect’ reversal of fossil fuel digging and burning, thus a ‘natural’ way to undo fossil fuel CO_2_ emissions.

#### Wood Vault in practice: prototype Version 1.1 (burial mound: Tumulus)

**Fig. 7:**
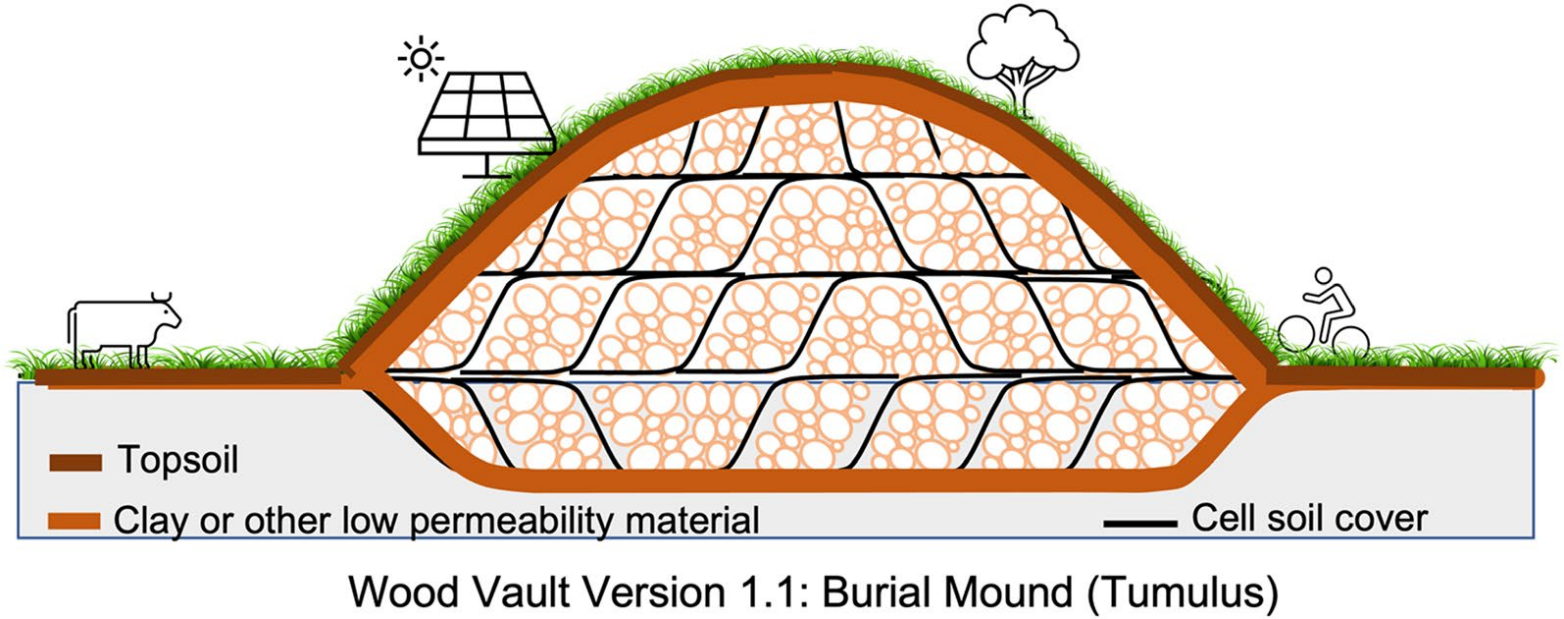
A basic Wood Vault unit partially aboveground, partially underground (Wood Vault Version 1.1: Burial Mound Tumulus). A prototype unit occupies 1 hectare of land (100 by 100 m, or size of two soccer/football fields), 5 m deep, 20 m tall. The unit is divided into cells. Each cell is sealed as soon as it is filled. After closing of the top at final enclosure, the original topsoil is put back, grass and shallow rooted trees can be allowed to grow, then used as park, grazing land, cropland, solar Farm, or combination of these. Reddish brown represents clay or clay-like low permeability soil or liner to ensure anaerobic condition, while dark brown represents backfilled topsoil

Our ‘poster-child’ Wood Vault is a burial mound, nicknamed Tumulus after the semi-underground human burial sites in the pre-historical Mediterranean region. What size of a large burial facility will be needed to accommodate this amount of wood? 100,000 tonnes of green biomass is about 100,000 cubic meters in volume. This volume of wood can be stored in a space of dimension 1 ha (100 m by 100 m; size of two soccer/football fields) with a trench 5 m deep, and a mound 20 m above ground. The air space of the structure is about 150,000 m^3^, after accounting for the tapered geometry of the mound. We assumed 1/3 of the volume is filling space occupied by clay sealant, water and soil backfill. The aspect ratio (height:length) of 20:100 gives a moderate slope. It can in principle be made even taller which would provide a nice vista point on flat land and recreational space if used as a park after sealing. It can also be made deeper at some additional excavation cost. Both can reduce the requirement of surface land area. Such a Wood Vault is illustrated in Fig. 7. This is Wood Vault Version 1.1 (Burial Mound: Tumulus).

We call this 1-hectare area a basic Wood Vault Unit (WVU, see below for more discussion), which is the size of two football/soccer fields. Obviously, any other practical sizes are also possible. It does not change the analysis below. On the timescale of a few months to a few years, the unit is filled and then enclosed at the top. Within each unit, multiple cells can be partially sealed with excavated soil more frequently, say each month, to further minimize decomposition before a complete seal off. Multiple Wood Vault units can be added over time and are independent of each other. At higher wood availability such as the rural harvesting case, the process can be done at higher frequency. This will be more effective for wood preservation and more cost-efficient.

It is also possible to use a similar size facility to bury wood collected from a larger area at the same wood availability per unit area. In this case, the facility can be filled to capacity earlier. The additional cost of transporting wood material over longer distances but within ~ 100 km may still be quite economical. The optimal size of the wood burial facility needs to strike a balance between size of wood source region and the cost of facility construction and transportation, depending on local circumstances.

#### Construction of a Wood Vault (Version 1.1 as example)

To accommodate wood material from a variety of sources such as urban wood residuals and forest residues, as well as woody biomass collected from a broader region, large-size Wood Vault can be built to handle the material efficiently. To construct such a facility (Fig. 7):

1. Firstly, estimate the sustainable rate of wood source within the region of interest.
2. Then select a suitable site with size commensurate to the estimated wood source and planned operation time horizon of the facility.
3. The suitability of the site is based on an assessment of site characteristics, including soil type, soil depth, soil profile, topography, hydrology, climate, environment, cost, ownership, and other relevant factors.
4. Collect wood to build up a stockpile for temporary storage before burial.
5. Then excavate soil to form a large trench/pit, with the soil laid on the side. The organic containing topsoil should be excavated first, separately from subsoil, in order to be put back on the surface later to minimize environmental impacts via land use change and provide nutrients and substrate for decomposers, as well as supporting vegetation regrowth on top. Wood is buried in the subsoil (B-horizon) or lower horizon, completely isolated from the biologically active topsoil above (Fig. 7).
6. Then divide the pit into multiple sections (cells). To minimize degradation before the closure of the cell, wood material is trucked over and laid down in the cell before moving on to the next cell. After it is fully filled, the cell is covered with a layer of soil and closed. Soil is compacted and allowed to settle to fill the gaps. The cell size is such that it can be filled, ideally in less than a few months of the first dump, and the shorter the better. The optimal cell size can be determined by balancing wood sourcing rate and engineering cost (larger cell will be more space efficient and cheaper per unit mass of wood stored).
7. It is useful to take into account the seasonal variations (cold vs. warm, or wet vs. dry) in carrying out different steps of operation. For instance, cutting wood at the end of the growing season after leaf fall will minimize nutrient lockdown as trees send their nutrients down to the rooting zone before shedding leaves. Logging/collection operations are best carried out in cold/dry season with frozen ground to minimize soil damage and compaction as well as to allow better machine maneuverability.
8. Wood material can be minimally pre-treated to extend the storage lifetime. For example, the treatment material can be contained inside a pond on site. Wood can be dipped into the pond, packed into easy-to-handle units after or before treatment. Another technique is to spray over the wood as the pile runs through a carwash like tunnel, especially on the cut surfaces. A cheaper option is to spray only the cut-ends of whole logs. Charring of the surface of the cut ends is yet another technique. The recommendation of minimal or no treatment is an economic consideration, not that treatment is not good for preservation.
9. Use the excavated local soil to cover the buried wood if it has low permeability such as clay. The permeability, measured in Darcy velocity, should be lower than 10^-8^ m s^-1^, and even better less than 10^-9^-10^-10^ m s^-1^. If clay is not available on site, source it from somewhere else. Fine silty and muddy soil may be used in case clay is not available, but wood durability may be lessened somewhat.
10. If covering soil is sourced from outside or the soil excavated is not reused for backfilling or covering, wood can be simply piled up without excavation (Version 1.2 below). This may be a preferred approach if the water table is very shallow and fluctuates significantly so that it’s difficult to keep the buried wood outside the fluctuating zone.
11. Where the topographic slope is significant, line with clay or possibly synthetic material the upslope-facing side to prevent water from moving laterally through the buried wood, in order to minimize episodic reoxygenation of the burial environment. The best method is, however, to seal the pile completely from all sides with clay.
12. If possible, the base of the pit should be either above the local water table at its highest level or below the lowest level to avoid fluctuating water–air boundary bringing in oxygen.
13. When considering all factors for site selection, if the water-level requirement (either completely above the seasonal highest water level or completely below the lowest seasonal level) cannot be satisfied, and the local soil does not have very low permeability, one can use clay to enclose the pit completely with sufficient thickness on all sides. The degree of anaerobic condition depends on clay thickness. We recommend a ballpark value of 0.3 to 1 m, which obviously depends on the quality/permeability of the clay.
14. After the facility is enclosed, grass or trees with shallow roots can be allowed to grow back, and the land can then be used as pasture for grazing animals, cropland, park, photovoltaic solar farms, or combinations of the above.
15. Some land settling over time may be inevitable. Carefully packing the filling material of wood and soil can minimize but is unlikely to eliminate settling. If wood eventually rots, even just partially, land settling will become significant, in which case the Wood Vault would have failed. Obviously, the purpose of Wood Vault is to ensure no or little decay over a long period of time. From an engineering point of view, the design lifetime is a key criterion, but because Wood Vault as well as its climate goal are novel concepts and we don’t have enough engineering data to quantify it, this is an area that needs to be further explored and better defined, in particular, in light of long-term climate goal of WHS as a thermostat (below).

#### Seven versions of Wood Vault

**Fig. 8:**
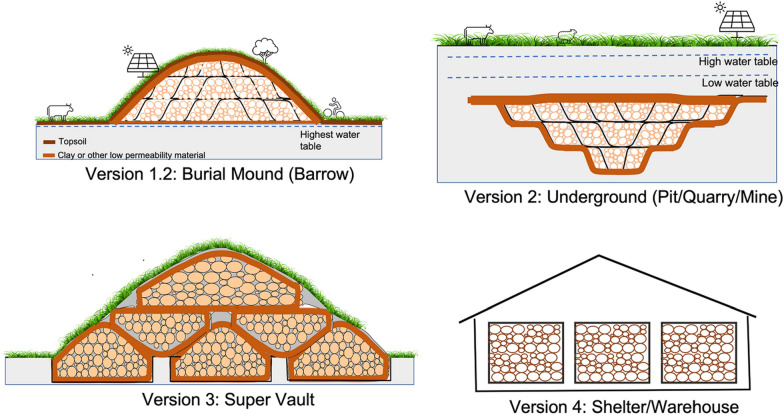
More versions of Wood Vault: Version 1.2: fully above-ground (Barrow); Version 2: fully underground (Pit/Quarry/Mine); Version 3: aboveground shelter/warehouse; Version 4: Stacked units (Super Vault): each sub-unit is one of Tumulus or Barrow

**Fig. 9:**
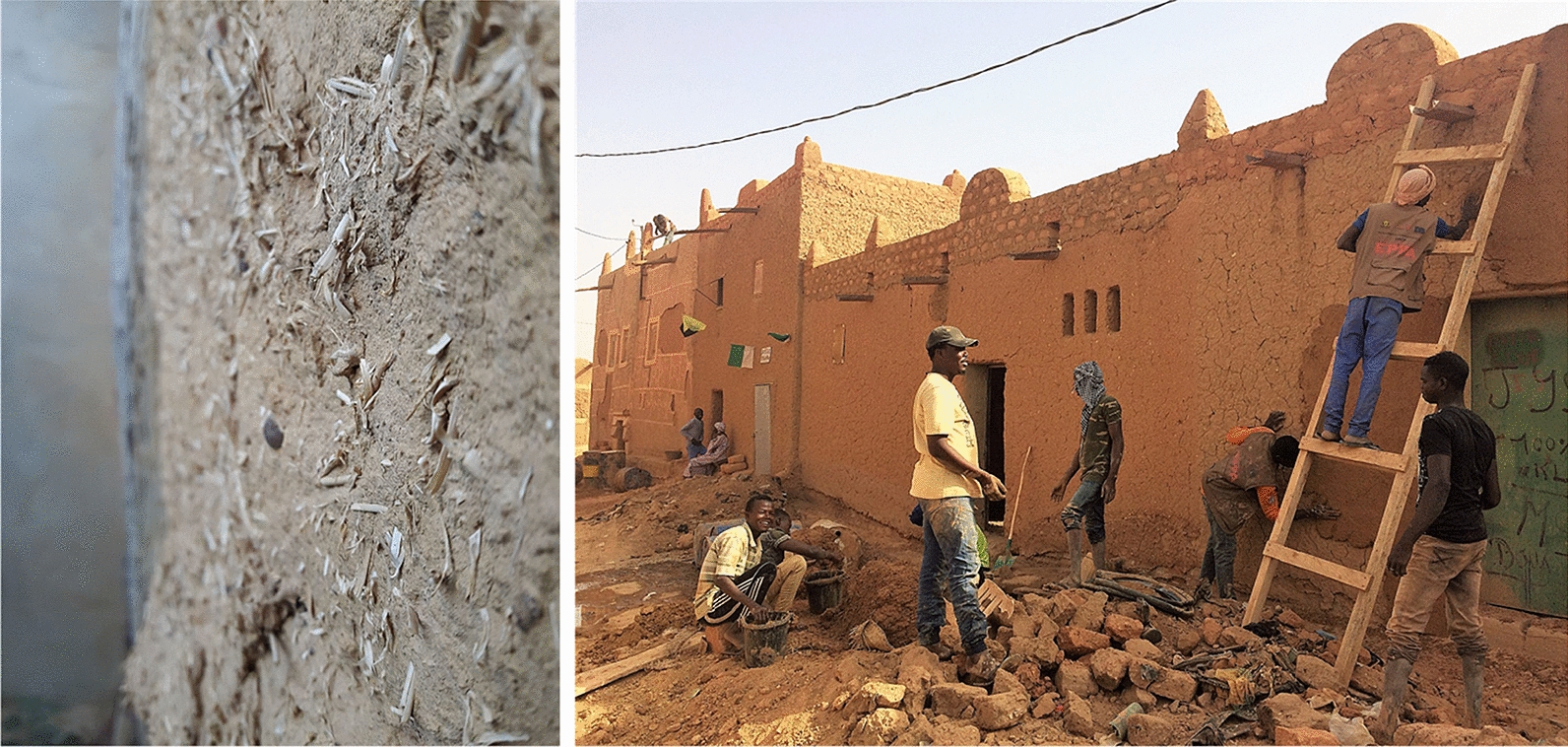
Adobe (Spanish) or cob (English), a traditional construction material/technique that mixes mud with fibrous material such as straw. Adobe can be an excellent choice for aboveground versions Wood Vault, for example, Shelter, DesertVault, or aboveground Baby Vault. Left: adobe wall in Palencia, Spain; Right: Maintenance of historic architecture in Agadez, Niger, by adding a new layer of mud rendering. Photo source: Wikipedia

Other versions of Wood Vault construction are also proposed (Fig. 8):

- Wood Vault Version 1.2: Fully aboveground (Burial Mound: Barrow).
- Wood Vault Version 2: Fully underground (V2.1: Pit, V2.2: Quarry, V2.3: Mine). Filling exhausted quarry or mine with wood provides a co-benefit for mine remediation.
- Wood Vault Version 3: Stacked multiple units (Super Vault). This will be more land use efficient.
- Wood Vault Version 4: Shelter/Warehouse. Woody biomass is stored in shelters built to keep out animals and insects. The shelters can be made of a variety of construction material, including wood itself. The wall can be made with traditional method of straw-mud or mudbrick (known as adobe in Spanish and cob in English; Fig. 9), sheltered from rain on top. Mud can usually be locally sourced. It is highly effective at fire prevention as well as acting as an insect/animal barrier. Multiple stockpiles can be further put inside a large warehouse, either open or closed. Slow wind erosion on the order of decades to centuries can be countered with some maintenance. Each sub-unit is a Baby Vault. Each sub-unit can be sealed airtight with adobe. Another option is not to seal off the wood piles, but have it ventilated naturally or with an engineered system. In this case, many more issues need to be resolved because it’s not anaerobic.

We use Tumulus and Barrow to distinguish Version 1.1 from Version 1.2 according to whether the Vault is partially belowground. In Archaeology, the term Tumulus, with its Latin origin, is commonly used to describe burial mounds in the Mediterranean region where they are typically built partially below ground, such as the Etruscan tombs in central Italy. In contrast, the term Barrow can be traced to a southern English dialect. The English tombs as well as northern European burial mounds tend to be built fully above ground. We hypothesize that this is due to the wet climate and high water table in these regions, while the drier Mediterranean climate allows underground burial. This distinction is more than just terminology. Indeed, it has important engineering implications for the construction of Wood Vault for wood preservation. A fluctuating water table leads to aeration and oxygenation, thus not conducive for maintaining anaerobic condition in a Wood Vault. Thus, in wet regions, the fully above ground version (Barrow) is better than Tumulus, though sufficient clay sealing can still make Tumulus version work. This is a critical factor in our design of several versions of Wood Vault.

The construction of these other versions may differ somewhat from Version 1.1 (Tumulus). For instance, the underground version (Version 2) would work well on low-lying area with high water table, where the wood can be buried completely underground below the lowest level of fluctuating water table.

1. Such places are typically low-lying land with little lateral transport, so sealing requirement may not need to be as stringent but care needs to be taken as we are talking about a very long time scale. These places also tend to have silty soil as they are often formed by alluvial/fluvial sedimentation which already has low permeability.
2. In the case of a valley or other sloping topography, there may be strong one directional underground water flow. In this case, the upslope direction should be lined with clay or synthetic liner to minimize water flow in the burial chambers.
3. Similar methods can be applied to bury wood in abandoned mines or quarries which involves little digging.

Burying the wood completely below ground will require more excavation, thus somewhat higher costs. The excavated soil may be sold to compensate for the excavation cost, depending on the market. In the case of utilizing an exhausted mine or quarry, there is little cost of excavation, and additionally contains many environmental and social co-benefits.

Compared to the underground versions, aboveground Wood Vaults would require more maintenance. For Vaults with an open woodpile, a key factor would be fire prevention. Traditional construction material such as adobe/cob (mud-straw brick wall) is an excellent choice because it is locally sourced and low-cost. An adobe enclosure of woody biomass can last for decades or longer without major maintenance (Fig. 9). Synthetic material can also be used. In wetter climate, a roof will be needed for rain sheltering. Another concern is animal burrowing or wind erosion, both can be repaired in time with low-cost.

**Fig. 10:**
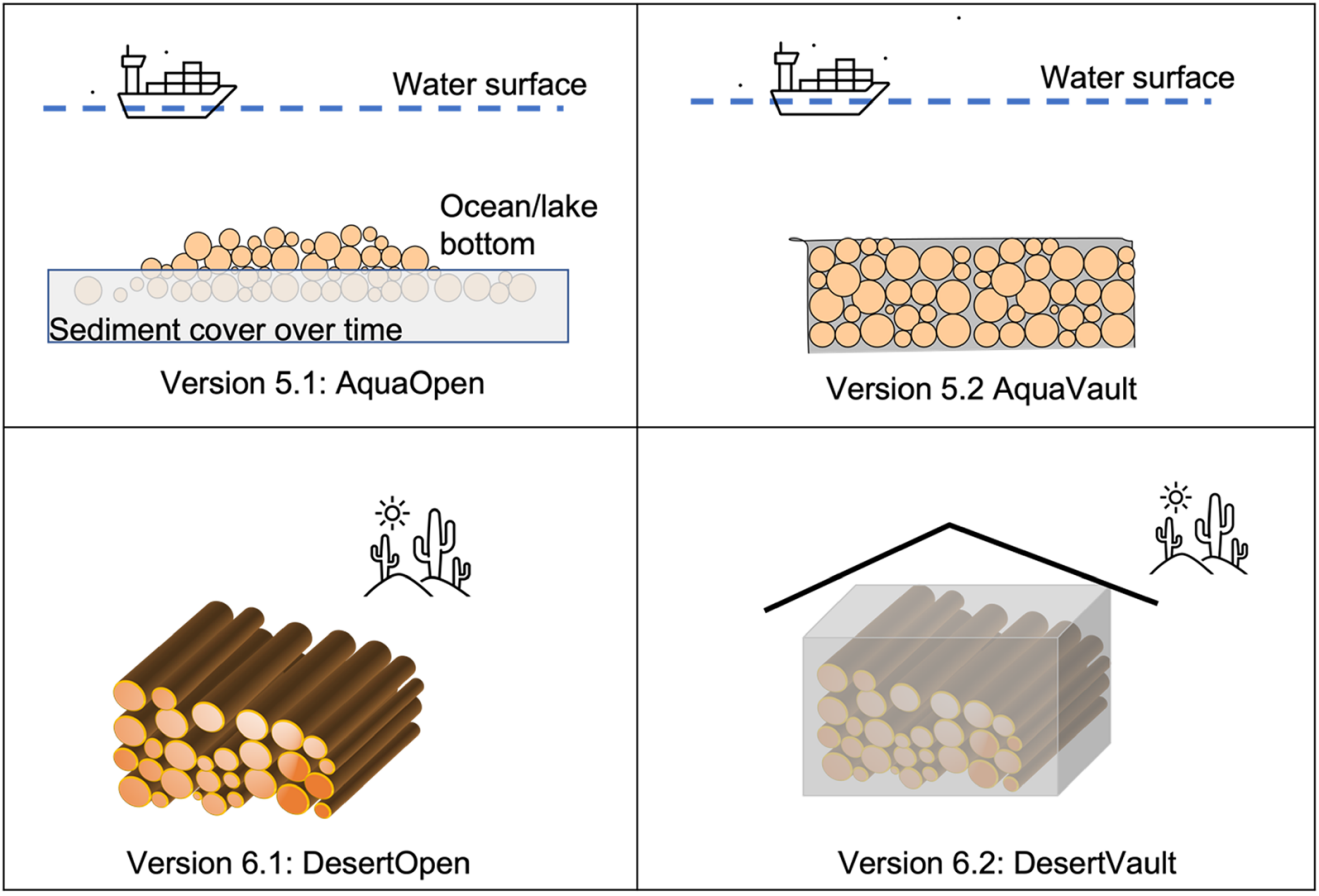
More Wood Vault versions. Version 5.1 AquaOpen: submerged underwater, open without protection. Version 5.2 AquaVault: submerged underwater with protection. Version 6.1 DesertOpen: stockpiled in dry condition (desert) without protection. Version 6.2 DesertVault: stockpiled in dry condition with protection

It is also possible to store wood in perpetually wet (submerged under water) or dry (desert) conditions [3] (Fig. 10), which leads to our Versions 5 and 6:

- Version 5.1: AquaOpen. Wood logs are barged over to water bodies with low-oxygen bottom water such as the Black Sea or the Great Lakes and dropped to the bottom. Ballasts (weights) may be needed to sink the logs, but eventually the logs will remain sunk after saturation with water. It is possible that some dried logs may be resin-sealed so that water cannot penetrate the wood so some research is needed here. A good technique is pre-soaking the logs. Not all water bodies are suitable because bottom organisms such as marine borers and bacteria can attack wood. These waters are not completely devoid of oxygen due to mixing, no matter how weak it is, but the biological activities may be slow enough such that sediments can cover the logs before significant wood decay occurs. Once trapped in the silty sediments, the wood should stay semi-permanently. Examples of wood preservation include the well-preserved Roman and Greek wooden ships at the bottom of the Black Sea, the Swedish Vasa warship at the bottom of Stockholm harbor, and the Neolithic village wooden artifacts at the bottom of Lago di Bracciano in Central Italy, as well as the newly discovered Endurance ship of Ernest Shackleton preserved in near-perfect condition 107 years after sank to the bottom of the Weddell Sea, Antarctica.
- Version 5.2: AquaVault. Bundles of wood logs are wrapped inside a lining material, for example plastic or rubber, that is resistant to attack from bottom water dwelling organisms, then are sunk to the bottom. The wrap does not need to be completely waterproof. Similarly to a diving wetsuit, as long as the wrap maintains integrity, the water inside the wrap and between the wood logs will be stagnant enough to maintain low enough oxygen concentrations to prevent bacteria and the wrap itself will deter marine borers. Burying logs in bogs and wetland can also be classified in this category, though it can equally fit in underground burial category.
- Version 6.1: DesertOpen. Piles of wood logs are left open in the desert. Dry air and wind minimize decay. Fire is a potential hazard. Having relatively small wood piles separated from each other can minimize loss in the event of fire. Piling the logs in a way such that it is naturally ventilated can significantly extend the life of preservation.
- Version 6.2: DesertVault. Wood piles are protected with synthetic material, or the traditional methods of straw-mud walls or mudbrick (adobe), sheltered from occasional rain on top. Mud can usually be locally sourced. It is highly effective at fire prevention as well as acting as an insect/animal barrier. Slow wind erosion on the order of decades to centuries can be reinforced with some maintenance. In desert regions, wood sourcing is more of a limitation as forests are mostly in the mountains with modest productivity and many other desired uses. Thinning for fire risk reduction may be a major opportunistic source. Transportation cost is another limiting factor, but there should still be good opportunities for relatively in-situ storage. DesertVault bears similarity with Version 4 (Shelter/Warehouse), but it can be much simpler because the dry desert climate poses significantly lower risk to wood preservation. There are two options of airtight sealing vs non-airtight, whose pros and cons need to be further evaluated though our current inclination is for airtight because we think climate goal needs a guaranteed lifetime much longer than 100 years, which is a commonly stated goal in the current Carbon Dioxide Removal and Carbon Sequestration communities (See below discussion on WHS as climate thermostat). Same consideration applies to Shelter (above) and Baby Vault (below).

**Fig. 11:**
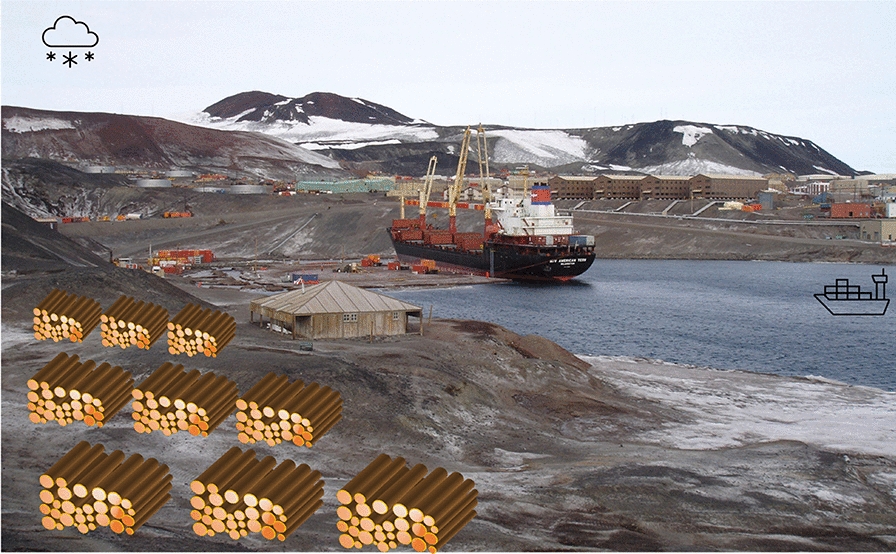
Wood Vault Version 7: FreezeVault. Wood piles are stored in cold regions such as Antarctica. Illustration is conceptual, and it does not necessarily imply actual site or wood storage details. Background photo: US McMurdo Station, Antarctica ( source: Wikipedia)

Finally, we propose Version 7, named FreezeVault, where wood piles are stored in cold regions such as Antarctica (Fig. 11), ideally in locations perpetually below freezing, but occasional short-term warmth should not be a problem. For safety, we should also take into account possible future warming. The wood piles are open in a natural freezer-like environment, which we still call conceptually a Vault because even though  there is no physical protection to construct, it is a natural place we move the wood to. The transportation, placement etc. still consist of engineering effort. Transportation cost dictates that it needs to be in the coastal region, perhaps 1–2 m above sea-level in case of future sea-level rise, though moving the piles upland by 1–2 m are totally feasible should it become necessary in the future. The wood piles should not be placed on fast moving glaciers.

Transportation cost for some versions above may be a concern, such as water bodies and Antarctica as wood source may be far away from the storage site. However, wood logs can be rafted down water ways, and barges or bulkers can be used for long-distance transportation of bundled wood piles. The cost should be quite manageable given the state of modern shipping technology.

Even though we have focused on local wood sourcing here, we do not exclude possibilities of transporting wood long-distance via waterway or railway that may be sufficiently economical and carbon efficient.

#### Land use after enclosure and ensuring the longevity of Wood Vault

Another attractive aspect of Wood Vault is the value for later land use. Unlike a landfill for municipal waste disposal, where leachate, odor and methane gas from chemical and biological activities are of major environmental concern, a Wood Vault is clean, stable and safe.

After the topsoil from excavation is backfilled after enclosure, grass and certain types of trees can be allowed to regrow. It can be used as pastureland or other agricultural use, or recreational use such as parks (Fig. 4). Yet another attractive possibility is to use it to install solar panels to generate electricity, or agrivoltaics (combination of above). We expect good safety after post-burial soil settlement for low-weight buildings such as warehouses and animal barns, but as a precaution, it may not be ideal for constructing houses and some other building types. This is a precaution for the rare case where the burial was not done properly or unforeseen geological activities occur because a well-constructed Wood Vault should be as solid as normal ground after a period of soil settlement.

Another key step is ensuring the legal permanence of the Wood Vault. Some legal framework must be set up to ensure that the buried wood isn’t disturbed on a century timescale or longer, even if the surface is utilized for other purposes or even sold to a new owner. There are several potential methods to ensure this. The first is to set up a legal entity to take control of the land containing the Wood Vault and be in charge of its maintenance and upkeep, possibly making use of the surface but with a primary objective of ensuring sequestered wood permanence. While this may seem simple, it can add other costs and ensuring the century long survival of such an entity is a significant challenge.

Another option is to set up a conservation easement on the land. A conservation easement restricts what land can and can’t be used for, with common limits being natural land, agriculture, or sustainable forestry, none of which should interfere with the permanence of sequestered wood. Easements are usually set up on a case-by-case basis, so the preservation of wood could be made an explicit tenet of the easement. Thus, even if the property is eventually sold, the permanence of sequestered wood is legally guaranteed. This approach is much more likely to survive the century scale required for sequestration projects. The easement also has the effect of lowering land values and property tax burdens, reducing expenses if the sequestration entity decides to actively run or manage the Wood Vault after the final burial and capping of sequestered wood. Additionally, buying insurance for possible loss of the stored wood due to unforeseen circumstances would also be useful, but this is an after-fact measure, not a design objective.

### The economics of Wood Vault

#### Wood Vault with wood residuals as source

The cost of a Wood Vault facility includes land purchase, construction, and operation. Suitable land in the suburban US East, such as Maryland, ranges in value from $10,000 to $40,000 per acre, or $25,000–$100,000 per hectare, but can be as low as $2000 per acre in more remote regions. Construction cost will be dominated by excavation and sourcing of clay (should local soil not suitable), estimated at a unit cost of $4/m^3^ and $15/m^3^, respectively [10]. Work and quality control (QC) will also be a large budget item. Transportation of wood from source to the facility is assumed at a rate of $5/ton for a 25-mile haul [6]. This cost would be zero, or even negative if the facility acts as a waste disposal station which typically is paid for a tipping fee to accept urban solid waste.

Altogether, the estimated cost for the 1 ha Wood Vault unit will be $1.2–1.8 million for storing 100,000 tCO_2_ in 1 year, at a price of $12–18/tCO_2_ sequestered (Table 3).

**Table 3:** Recurrent cost each year for a Wood Vault unit

Carbon stored per year	total cost per unit cell per year	Land purchase	Construction	Operation (staff, management, monitoring)	Transportation	Size of a unit cell
100,000 tCO_2_^+^	$1.2–1.8 million	$25–100K	$700K^++^	$500K	$0–500K^+++^ ($5/ton-25mi)	1 ha (2.5 acre) 20 m high 5 m deep

^+^Collected from wood residuals in a surrounding region of 50 km by 50 km

^++^Excavation = $200K ($4/m^3^ × 50,000m^3^); material/clay = $300K ($15/m^3^ × 10,000m^3^); work/QC = $200K

^+++^Transportation would be at no cost to the facility if accepting wood

10 such Wood Vault units, as in the urban wood residual collection case after 10 years, or over a larger area in one year, would sequester 1 MtCO_2_. The total cost, now including up-front and other one-time costs will be $13–22 million (Table 4). The cost of sequestered carbon is $13–22/tCO_2_.

**Table 4:** Economics of the facility after filling 10 Wood Vault units (assuming urban wood residual collection as the only source)

Total carbon sequestered	Market value at an assumed price $50/tCO_2_	Total cost	Recurrent cost	One-time cost (Equipment, building)	Land purchase
1 MtCO_2_	$50M	$13-22M ($13–22/tCO_2_)	$12–18M	$1M	$1–4M (40 ha) ^+^

It also applies to collecting wood at higher rate but in shorter time. Cost in millions of USD

^+^ Land is purchased up front and is larger than Wood Vault area: 40 ha (100 acre) area is assumed at a cost of $1–4M ($10–40K/acre, and cost less in remote region), with 10 ha used for Wood Vault, and extra 30 ha for operation or future use

If the wood is sourced from residues from fuel treatment, logging, or forest clearing from commercial development, all incurring little extra cost, the total wood availability will be much higher. Similarly, if wood is sourced from larger area than 2500 km^2^, a larger source will be available at somewhat higher transportation cost. In such cases, the facility can achieve higher carbon sequestration rate in shorter amount of time.

#### Wood Vault with harvested wood source and transportation

The above example assumes a central facility that collects only urban wood residuals. The cost estimate can serve as a basis for other types of storage and wood collection methods. For example, should wood be sourced from managed forests in the surrounding region, additional cost of raw material, harvest, and transportation should be included. Assuming a stumpage price of $5/tonne, harvest and transportation of $15/ton [3, 11], this adds $20 to the basic scenario above ($13–22/tonne) whose high range includes transportation. Also importantly, land and operation cost would be significantly lower if co-siting with an existing landfill, but this scenario is not used in our estimates here.

Altogether, we give a cost range of $10–50/tCO_2_ with a mid-value of $30/tCO_2_ for carbon sequestration in large Wood Vault storage facilities. The large range arises mostly from the following factors: (1) whether the wood source is ‘waste’ (at little or no cost) or harvested (higher cost), (2) transportation distance, (3) local soil and environmental condition and (4) land cost of the storage facility. Our estimate does not include transaction cost in a carbon market.

### Put it all together: operational considerations

#### Operation

**Fig. 12:**
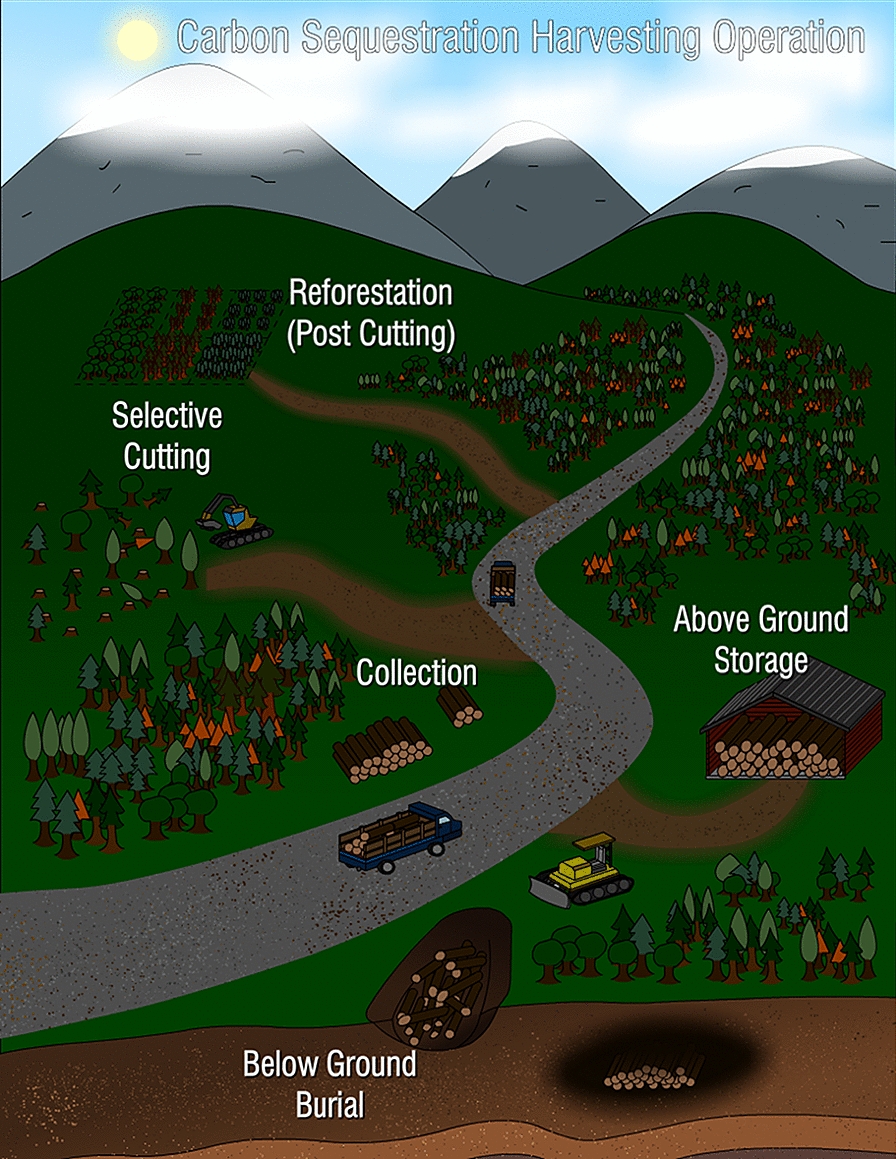
A vision of Wood Harvesting and Storage operation, showing how an operator can move crew and machinery around a large region from one plot to the next. The burial can be done in-situ or the wood can be transported outside the region to a large facility. Illustration by Wesley Tse of the Gemstone Carbon Sinks Team

To summarize, we illustrate in Fig. 12 an envisioned operation. The operator, say a ‘WHS company’ crew with machinery goes around, harvesting/collecting wood, then bring the wood to burial sites or stockpile before burial operation. A variety of specific operation styles can be envisioned based on wood availability (Type-A vs Type-B), burial site (large facility further away, or small facility mostly in-situ).

#### A unit for Wood Vault: implication for the scale of operation

To facilitate macro-scale planning, bookkeeping and carbon accounting, we propose a unit for Wood Vault, simply named Wood Vault Unit (WVU). One WVU is defined as a semi-permanent carbon storage containing 100,000 tCO_2_ equivalent of wood.

This simple definition provides an intuitive linkage between carbon accounting and the physical size of a Wood Vault, among other potential usages. It has the following attributes:

- A Wood Vault of 1 WVU stores 100,000 tCO_2_ equivalent of wood, by definition.
- A Wood Vault of 1 WVU contains an effective wood volume of 100,000 m^3^, occupying 1 hectare (100 m by 100 m, the size of two soccer/football fields) surface area, with an effective height/depth of 10 m, but actual height/depth of 20–30 m to account for the space in between the woody biomass occupied by backfill material, water/air and the geometry of the burial mound. These numbers are only approximate, and a design can be wider and lower where land availability is not a tight constraint.

**Fig. 13:**
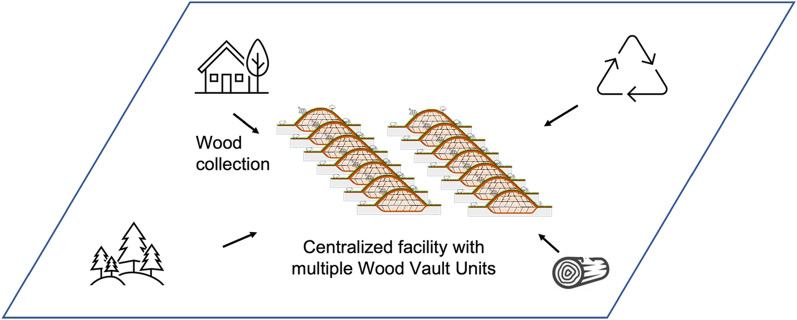
A centralized facility that hosts multiple Wood Vault Units (WVUs)

For an application example, a target of 1 MtCO_2_ y^−1^ sequestration rate needs to construct 10 WVUs per year (1,000,000/100,000 = 10), thus requires 10 ha of land surface. Up to 2–3 times more storage capacity is possible if the units are stacked vertically (Super Vault). Because a 1 MtCO_2_ y^−1^ rate can be satisfied with a wood sourcing scenario of medium harvest intensity from 2 counties in the eastern US (Table 1), which we assume to be a likely scenario in the next few decades, a 300 ha facility will store 30 MtCO_2_ in 300 WVUs, after 30 years of operation with wood sustainably sourced from the surrounding 2 counties (Fig. 13). We note that 300 ha is the size of a medium-size landfill. For example, the Brown Station Landfill at Prince George’s County, Maryland occupies 850 acre (340 hectare).

The implication of the above analysis is that a Wood Vault facility can be deployed sustainably at a scale an order of magnitude smaller than current landfill operations. Should the local government be the sole operator of all the Wood Vaults, simply co-siting with current landfills and sharing existing machinery and workforce will be enough to utilize its sustainable wood source, with the great benefit of minimum additional infrastructure, and high cost-efficiency. Should private entities such as individual farmers, forest owners, or corporations develop and operate the facility, the overall cost may be higher, but it should still be viable even with the current carbon market price.

For a target of 1 GtCO_2_ y^−1^ sequestration, we need 1000 such Wood Vault facilities, compared to more than 6000 operational landfills in the US. Since the above assumed medium harvest scenario that provides 1 MtCO_2_ y^−1^ available woody biomass is suitable for typical forested area and US forest area 3 Mkm^2^ is about 1/3 of the country, we estimate that the US alone can contribute 1 GtCO_2_ y^−1^ sequestration rate by operating a network of Wood Vault facilities on a scale 10–20% of current operating landfill facilities.

#### Baby Vault: the small operator model and an opportunity for technology innovation

Our analysis so far has assumed a ‘developed country model’ in a ‘big operator mode’ for its efficiency and economy of scale. In particular, we assumed the availability of machinery needed for logging, transportation, excavation, and burial on a large facility, supported by a corporate-style business operation. However, in many developing countries such as in Africa, the southern Amazon, or Southeast Asia where there are great opportunities for wood sourcing, including areas with potential for reforestation, this is simply not possible, at least not in the near term.

We propose a ‘small operator model’, as envisioned originally by Zeng [3]. In this operation mode, wood is sourced and stored in-situ. The relatively small wood collection area means that the stream of wood availability is also small. With manual and animal power, wood transportation quickly becomes prohibitive at longer distance. This offers the opportunity for innovative technologies such as solar-powered all-terrain robots that can haul logs, replacing traditional labor done by horses and mules. Rechargeable small chainsaw is already widely available (NZ has one at home). Similarly, small and low-cost rechargeable excavator/backhoe can also play an important role. In general, a potential WHS carbon sequestration market can provide the impetus to bring down the cost of existing technology or stimulate new technology in forestry operation from cutting, delimbing, forwarding, as well as various aspects of Wood Vault construction.

**Fig. 14:**
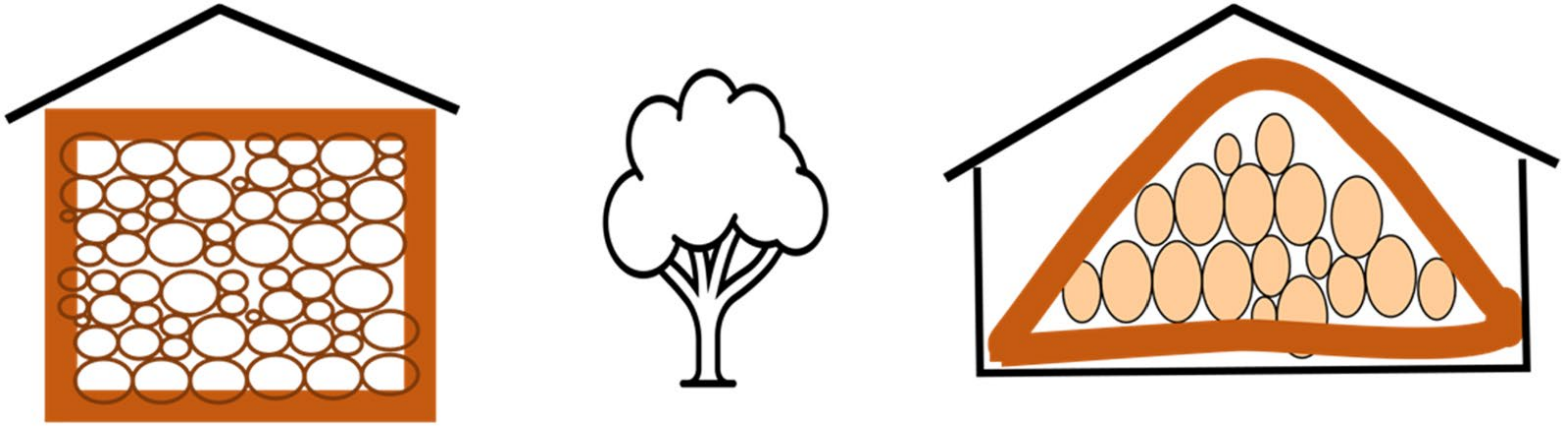
Baby Vault is a small shelter that can be built manually or with light machinery, using wood sourced from the immediate vicinity. The enclosure can be constructed with traditional adobe technique using local material of mud and straw. An underground version is also possible. Version Baby1 (Left) constitutes vertical walls that may be constructed before filling in logs, while Version Baby2 (Right) piles up logs naturally first before covering with mud or clay

Because wood stockpile permits only temporary storage before significant degradation occurs and now with wood collection from immediate vicinity, wood is best buried/stored in a relatively small Wood Vault, which we will call ‘Baby Vault’. Baby Vault is not necessarily a new type of Wood Vault, but just a smaller version of, for example, Tumulus (Version 1.1), Barrow (Version 1.2) or Shelter (Version 4). The Shelter-style Wood Vault (Version 4 or Version 6.2 DesertVault) seems ideal as Baby Vault for developing countries, run by individual farmers, co-operatives or other small operators (Fig. 14). It does not require digging which is labor intensive without machine. Piling the logs above ground is much more practical, though the height may be limited, thus using somewhat more land-surface area compared to a large burial mound.

For Shelter-style Baby Vault, an adobe/cob enclosure is particularly attractive because of the easily sourced local material and the practical experience of building/maintaining such structures across many human cultures. A straw-mud walled structure can be built, wood can be placed in it over a suitable period of time, then finally enclosed on the open side and on top which is sheltered with a thatched roof in wet climate (Fig. 14). The walls need to be sufficiently thick to be both structurally sound and prevent erosion and animal intrusion. Vines may grow which can be cut back or left alone, though trees should not be allowed on top. Long-term maintenance is needed but is expected to be minimal if the Baby Vault is constructed properly. We also note that, Baby Vault is not limited to developing countries, and indeed we see great potential in developed countries as well where an individual land owner can carry it out independently.

In-situ storage on the forest floor, near the logging landing site, or on the roadside where wood is harvested has the advantage of minimizing transportation cost and other benefits [3]. Indeed, Zeng [3] estimated a cost of $14/tCO_2_ for such in-situ operation. A disadvantage is the cost of moving machinery to the site and other overhead costs of each operation. It may be most efficiently carried out by an operator who continually goes around a certain large area consisting of many smaller sites with multiple ownerships, moving from one plot to the next on the time scales of days to weeks to minimize cost of transporting machine. The crew can return to the same site after some years for another harvest (Fig. 13). Materials and machinery can be planned out and used most efficiently this way. In practice, there is likely going to be a continuous spectrum of possibilities between small-scale in-situ burial and large-scale facilities.

#### Process flow diagram: a Nature-Engineering combo method

**Fig. 15:**
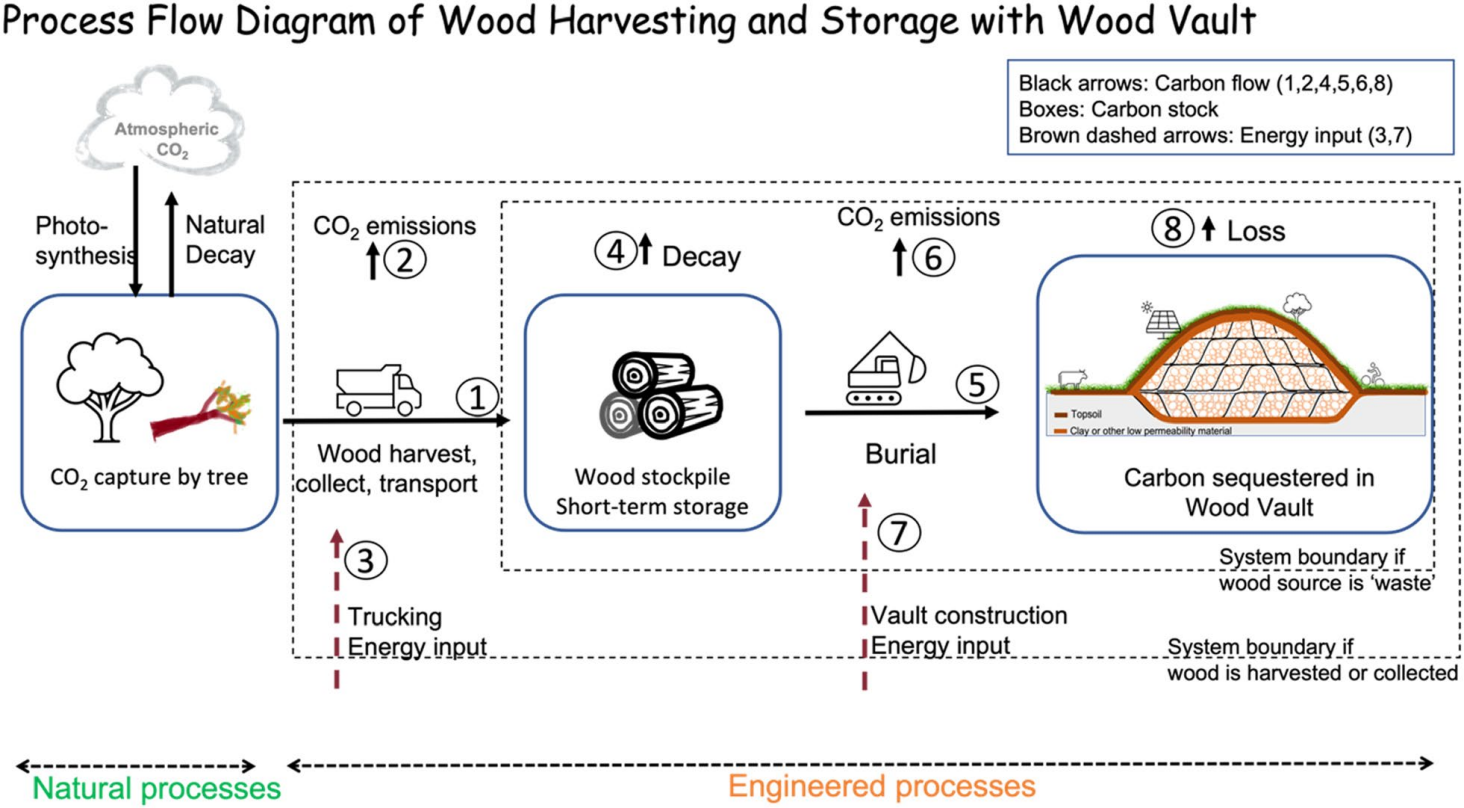
Process flow diagram (PFD) of Wood Harvesting and Storage with Wood Vault. The Natural Processes and the Engineered processes are clearly separated, defining the engineering system boundary. The two types of wood sourcing (wood residual reception or Type-A vs. harvesting or Type-B) correspond to somewhat different system boundaries. WHS takes advantage of ‘free’ photosynthesis and a simple engineering process to minimize wood decay, thus offering an effective, efficient, and low-cost Nature-Engineering combo solution for semi-permanent carbon sequestration

To close the loop of a full-scale operation from wood sourcing to burial, we show the process flow diagram (PFD) of WHS with a central storage facility Wood Vault (Fig. 15). The system boundary depends on whether the wood sourcing is ‘waste’ reception (Type-A) or harvested/transported (Type-B). It is also possible to have an operation somewhere in between Type-A and Type-B, for example, harvesting is already done, but the Wood Vault operator needs to collect wood from a remote site. Also, a wood stockpile for short-term temporary storage is often needed to strike the balance of efficiency/cost and minimizing decay before burial. The diagram lends itself naturally to full life cycle analysis (LCA) should the project-specific data input be provided for carbon and energy flows 1–8.

The fact that CO_2_ capture via natural photosynthesis is ‘free’, an evolutionary wonder from an ecological perspective, puts this process outside the engineering system boundary. This is a great advantage of WHS relative to purely engineered methods such as Direct Air Capture (DAC) where CO_2_ capture accounts for the lion's share of cost and energy input. Of course, this advantage is shared by all nature-based methods, thus their popularity. However, the main shortcoming of most nature-based methods is that the permanence of such carbon sinks is generally too short compared to the climate change time scale of hundreds of years. This shortcoming is overcome in WHS by burying wood underground or other means to ensure semi-permanent preservation. This requires dedicated facility carefully engineered to prevent decomposition and an operational process flow that is practical, efficient, and low cost. In summary, WHS is a unique hybrid Nature-Engineering method that combines the advantages of Nature and human intervention, each of which is inadequate for solving the climate problem if acting alone.

#### How to do it right: Hit the road with model projects

The best method to ensure the durability and semi-permanence of stored wood is to make sure the project is done right at the early stages of Wood Vault construction. If a Wood Vault is found faulty later, reinforcement is possible if the cost is low enough. This ‘reinforcibility’ should be an important criterion in determining the Wood Vault type of choice. For instance, a Wood Vault constructed in an adobe mud enclosure can be easily repaired should it crack. On the other hand, wood logs sunken to remote ocean depths are left completely to forces of nature as it would be too expensive to intervene at scale. Thus, knowing the processes that determine the timescale of a particular Wood Vault construction with our best knowledge, we can make the decision based on our climate goal (which is not necessarily clearly known on long timescales; see Sect. 5) will be critically important before we implement a specific type of Wood Vault at large scales. In this respect, the terrestrial based methods generally appear to be safer in the sense that they give us the opportunity to reinforce and modify in the future. To put it another way, higher standards will be needed for Wood Vaults that cannot be maintained and reinforced cost-efficiently.

We therefore wish to see the community to start as soon as possible a suite of ‘model projects’ that encompass the proposed Wood Vault types in representative environments, plus any other possible types we have not discussed here. In planning and conducting these model projects, we will gather interdisciplinary teams of experts and practitioners to figure out the best practical way of Vault construction that ensures durability for the specific environment at low-cost. These projects can serve as the ‘blueprints’ for world-wide implementation of WHS. Our current knowledge can already inform reasonable decision because there is no truly unknown science or technology in the WHS method, although the interdisciplinary knowledge base needs to be put together to tackle this problem effectively. Iterations will be needed to refine these blueprints. This is also where governments, academics and the scientific community can and should play a key role.

#### Monitoring and verification

**Fig. 16:**
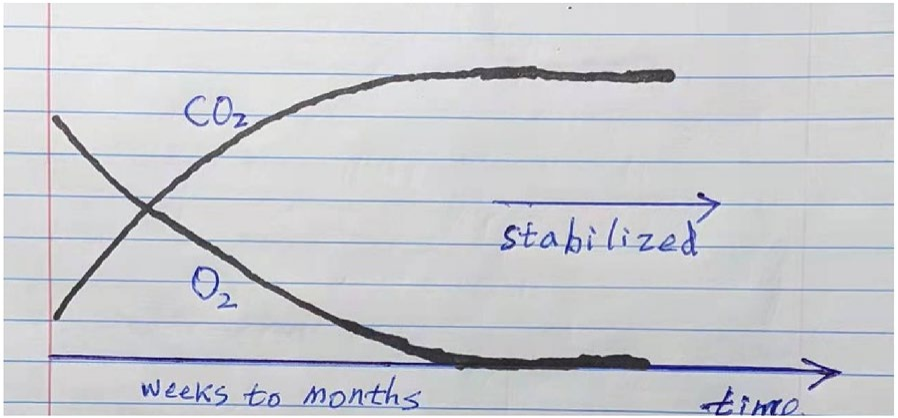
The expected changes over time of O_2_ and CO_2_ after the enclosure of a Wood Vault designed to produce anaerobic condition. The gas concentration levels stabilize after a few weeks to months as the Wood Vault enters a quasi-geological semi-permanent state of preservation

After the construction of a Wood Vault, monitoring and verification may be needed for getting carbon credits that can be traded in a carbon market, among other reasons. We propose the following steps:

- Use low-cost sensors to monitor the environment inside the burial chamber, including CO_2_, O_2_, CH_4_, pH, temperature, humidity, and water table. For anaerobic type of Wood Vaults, after Vault enclosure, we expect the initial oxygen level to drop to zero on the timescale of weeks to months, and CO_2_ to rise to a high level as a small amount of organic matter is consumed by fungi/bacteria/insects that are ubiquitously attached to or embedded in the original woody biomass (Fig. 16). For a well-constructed Wood Vault, we don’t expect CH_4_ release (see discussion in Sect. 5) so it should remain zero. Such low-cost sensor capability has been developed in recent years, including at our lab [12, 13].
- These sensors can be built using an Internet of Things (IoT) approach that transmits data to a remote server. Software can be designed to pick up anomalies in various data indicators, for example a rise in oxygen or methane, and action can be taken if practical. The information on the status of the burial chamber also provides data input to the carbon accounting system useful for calculating carbon credit discount (below).
- A powerful analog method can be used to predict future preservation by conducting ‘controlled’ experiments, similar to the unintended ‘natural’ experiments from archaeological and geological evidence (Sect. 5 below) that provide real-world evidence of long-term wood preservation. For selected sample projects, periodically collect samples directly from the Vault, observe and analyze the status of wood preservation. The corner or cell of the Vault can be enclosed again, leading to a slight modification to future preservation. We can then build predictive mechanistic models that include process understanding, validated by these ‘controlled’ experimental data as well as the data from the ‘natural’ experiments. This is akin to how we build predictive climate models that are validated by paleo and modern climate data. By the simple logic of ‘analog’, similarly constructed Vaults in similar environment will have similar degree of preservation as in a ‘control’ experiment. In this respect, a failed Wood Vault provides equally valuable information on how Not to construct a Wood Vault that way. The ‘model projects’ proposed above can best serve this analog purpose.

#### Full carbon accounting: sustainability and a role for data science

It is critical to conduct a full carbon accounting for the purpose of carbon credits. In the case of residual wood utilization, the stored carbon should be relative to a baseline that corresponds to waste wood decomposition over shorter time spans than the semi-permanent storage (Fig. 2). When wood is sourced directly from live forests, the accounting is more complex. Initially there is a loss of carbon after harvest. It is only after forest regrowth that the combined regrowth and stored carbon exceeds the baseline (Fig. 3). Ensuring the sustainability of wood sourcing, in light of many other current and possible uses of woody biomass, is a key to success for WHS.

**Fig. 17:**
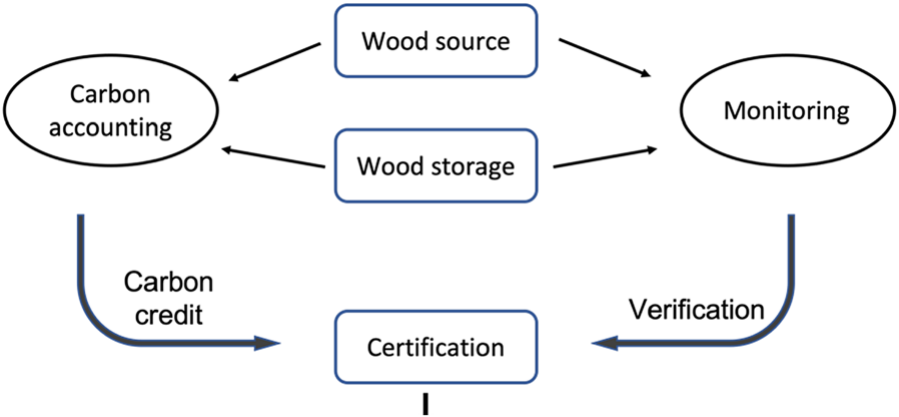
Monitoring and verification, carbon accounting, certification, carbon market for WHS

In a world where WHS is fully implemented, millions of Wood Vaults may be created. They need to be carefully monitored and maintained. Databases need to be built to track these Wood Vaults and their attributes, including: quantity (tCO_2_), quality (durability), sustainability (wood source, Vault construction environmental impact, etc.), location, ownership, and other details. Such information not only provides the foundation for carbon credit trading in carbon markets, but also the necessary information for monitoring and verification, ensuring sustainability, maintenance, insurance, unforeseen events, and potential future use. The information size will be small compared to the data we typically handle these days, but the quality, fairness and content matrix need to be worked out. The full process of monitoring and verification, carbon accounting, certification, carbon market for WHS is illustrated in Fig. 17.

### Durability and permanence of stored wood

#### Wood Vault is not landfill: biological, physical and chemical factors impacting wood degradation in a Wood Vault

**Fig. 18:**
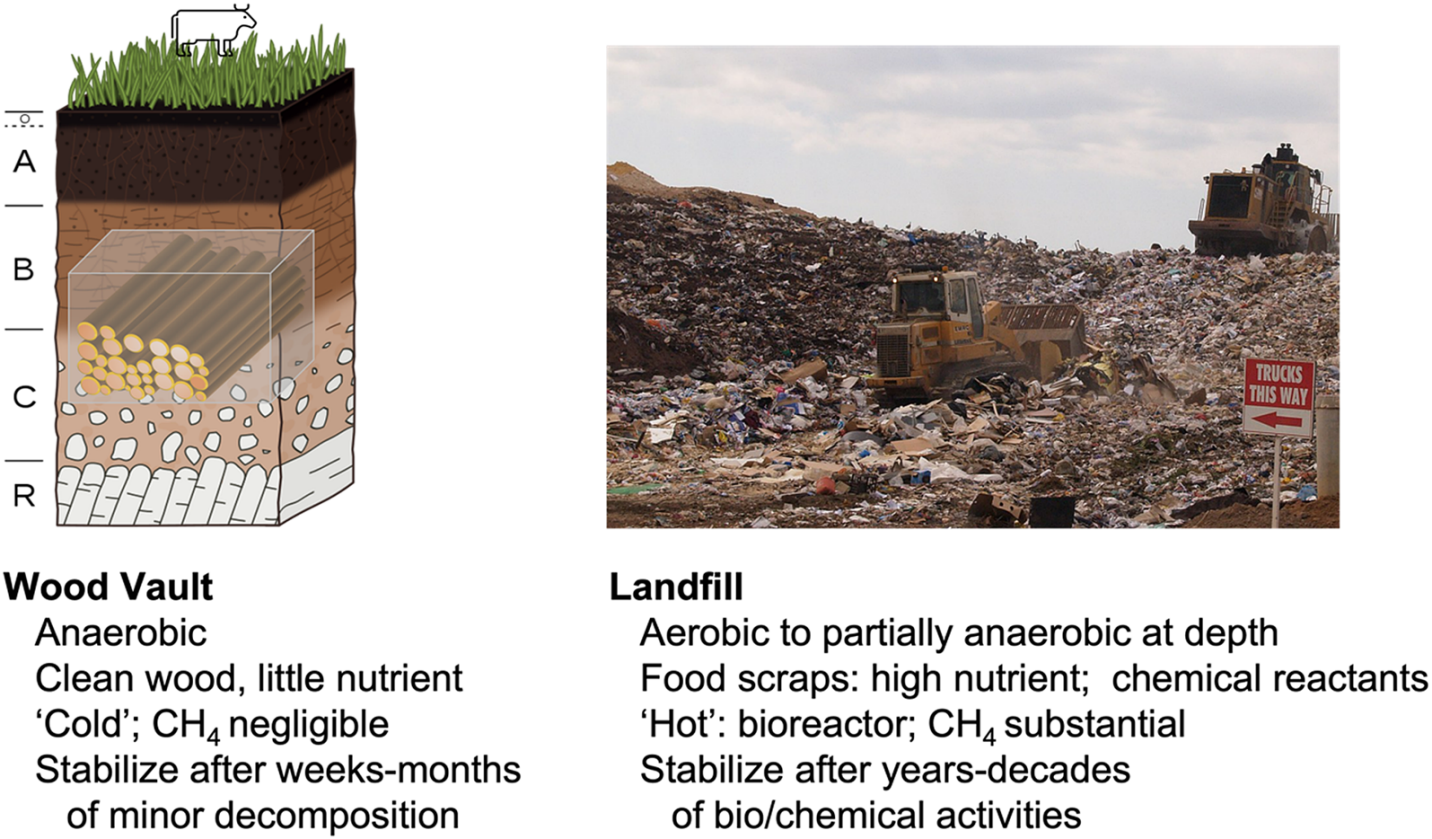
Wood Vault is fundamentally different from landfill. Photo credit: Ashley Felton

On the engineering side, the construction of a Wood Vault bears some similarity to a modern sanitary landfill as both involve digging and capping, so that we can use landfill data for estimating the economics of a Wood Vault. However, there are fundamental differences between a Wood Vault and a landfill, as illustrated in Fig. 18, including:

- The job of a landfill is to dispose of waste, including highly decomposable food scraps, reactive toxic chemicals, heavy metals that need to be carefully controlled and regulated. In contrast, a Wood Vault buries only clean natural vegetation which is valuable (for carbon sequestration now, with the option for biomass and bioenergy reserve in the future)
- The main objectives are the opposite of each other: a landfill is built to encourage fast decomposition and gas collection, while a Wood Vault is built to prevent decomposition altogether. This requires different design principles and different ways of material use.
- Methane (CH_4_), a potent greenhouse gas, is often generated in a landfill as methanogenic bacteria digest organic waste under partially anaerobic conditions. Such concern has been raised against the wood burial concept by citing what happens in a landfill [14]. However, this aspect of landfills does not apply to a Wood Vault. Furthermore, even in landfills, wood, unlike food, is often known to be well preserved, as shown by excavated wood samples from old landfills and modeling [15–17], even inside lab bio-reactors designed to encourage optimal bacterial activities [18, 19]. Thanks to such meticulous research, the IPCC has recently drastically lowered the emission factor of wood in landfills [20]. A precondition for CH_4_ generation in landfills is that there is sufficient concentration of nutrient such as food scrap to supply the substrate for anaerobic bacteria to thrive. This is not the case in a Wood Vault.
- Moreover, our standard Wood Vault design calls for total anaerobic condition in the sub-terranean burial chamber (with other designs having similar mechanisms to inhibit organism growth), reasoning that the lack of oxygen (O_2_) excludes activities of the main ‘wood-eating’ organisms such as fungi and insects. Then the main remaining risk to buried wood is anaerobic bacteria. Fortunately for our purpose, they are known to be unable to digest lignin, the glue-like layer that protects the cellulose structure [18], as evidenced by the difficulty of making cellulosic ethanol for biofuel where the challenge is the opposite: to get rid of lignin protection [21]. This explains why wood decomposition is slow in landfills. In a carefully designed and maintained Wood Vault, we expect the decomposition to drop to essentially zero after a short initial period during which any oxygen mixed into the wood-soil matrix initially is quickly consumed (Fig. 16).
- Decomposition of organic waste as well as chemical reactions by aluminum and other reactive materials in a landfill can lead to a ‘hot’ interior that typically reaches 40–50 °C (X. Wang, personal communication). The interior of such a landfill is essentially a ‘slow cooker’ that accelerates biological degradation and chemical reaction. Even in such ‘worst’ condition, wood is still often found to be reasonably well preserved (above). In contrast, in a Wood Vault, only ‘clean’ vegetation is buried, and there is little nutrient and substrate for bacteria to establish colonies at first place. A Wood Vault provides a ‘cold’ and fully capped environment without oxygen. This realization should be sufficient to relieve the concern of ‘smoldering wood’ or ‘smoldering garbage’ sometimes seen in open dump or poorly covered landfills. The key is to ‘do it right’.
- Whole wood logs are expected to have much higher durability than smaller pieces such as woodchips, as evidenced by multi-year to multi-decadal time scales (depending on the climate condition) needed to degrade whole dead trees on a forest floor, while smaller pieces such as twigs degrade much faster. The reasons are:
  - In both anaerobic or aerobic conditions, the degradation starts from surface and spreads inward very slowly. For a tree log, the spreading of fungal hyphae and bacteria is generally fastest along the cambium layer and inner bark where phloem contains highest concentration of nutrients. Secondly, they can spread along the vessels in the longitudinal direction or rays in the transverse direction from the cut ends or wounds. Certain tree species have high concentration of sap that prevents vessel penetration, thus minimizing this pathway of degradation.
  - In anaerobic condition, because the lignin layer covers the cellulose structure which is what anaerobic bacteria can attack, reduction in the wholeness of wood, leaves vulnerable entry points for bacteria to enter. Keeping the physical integrity thus enhances its durability.

Physical transformation of buried wood in a Wood Vault is unlikely due to the lack of light whose UV spectrum is known to degrade exposed exterior of wood structures. As for temperature, it is ‘cold’ (in comparison with an active landfill), same or slightly higher than ambient, and so is pressure. Should the wood preserve on geological timescales, it may be transformed (carbonized), but the carbon will still largely remain in place. Geological movement such as seismic activities could also potentially bring wood to surface or bring in oxygen. Such occurrence can be mitigated, but in practice, it is unlikely to be a major concern because of the rarity of such occurrence on the human-induced climate change timescale and the distributed nature of the WHS method.

A less clear aspect is potential chemical transformation. For example, a highly alkaline or highly acidic environment is known to damage wood (and many other things!). However, most natural soil environments do not deviate significantly from pH neutral. It is also possible that organic compounds such as terpenes in sapwood can be more easily modified by chemical or biological processes, especially in wet burial environments for tree species with less resin where water can infiltrate into wood vessels and leach out organic compounds inside. However, modification doesn’t necessarily convert the organic matter into CO_2_ which would subsequently escape back into the atmosphere. If conversion to CO_2_ indeed happens, albeit likely slowly, this fraction of carbon would be lost, but the main wood structural ligno-cellulose carbon should remain. In mineral-laden subterranean wet/aquatic environments, minerals would slowly infiltrate wood to seal off the vessels, likely leading to better preservation. While research will be needed to clarify these complexities, carbon accounting for credit can conservatively exclude carbon stored in such organic compounds which only accounts for a small fraction of wood carbon. Thus, the goal of preservation is not necessarily to keep the wood like fresh wood, but can permit transformation to a stable state that maintains wood integrity and keeps most of the carbon in place semi-permanently.

### Durability: lessons from archaeological and geological evidence

**Fig. 19:**
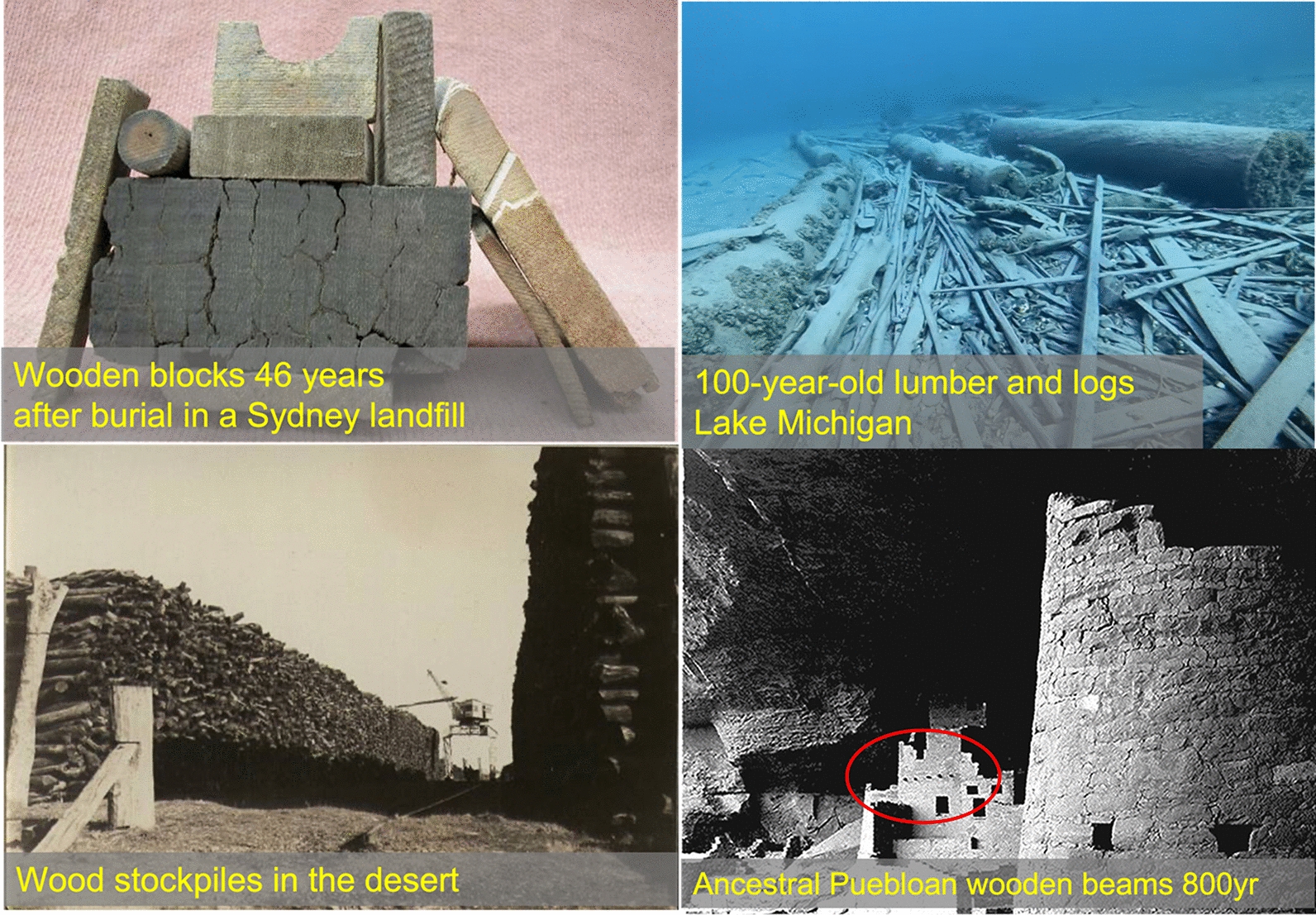
Preserved wood of human origin: decades to centuries. Photo credits: F. Ximenes, Chris Roxburgh, unknown via Matt Pearson, Ansel Adams via National Archives and Records Administration

**Fig. 20:**
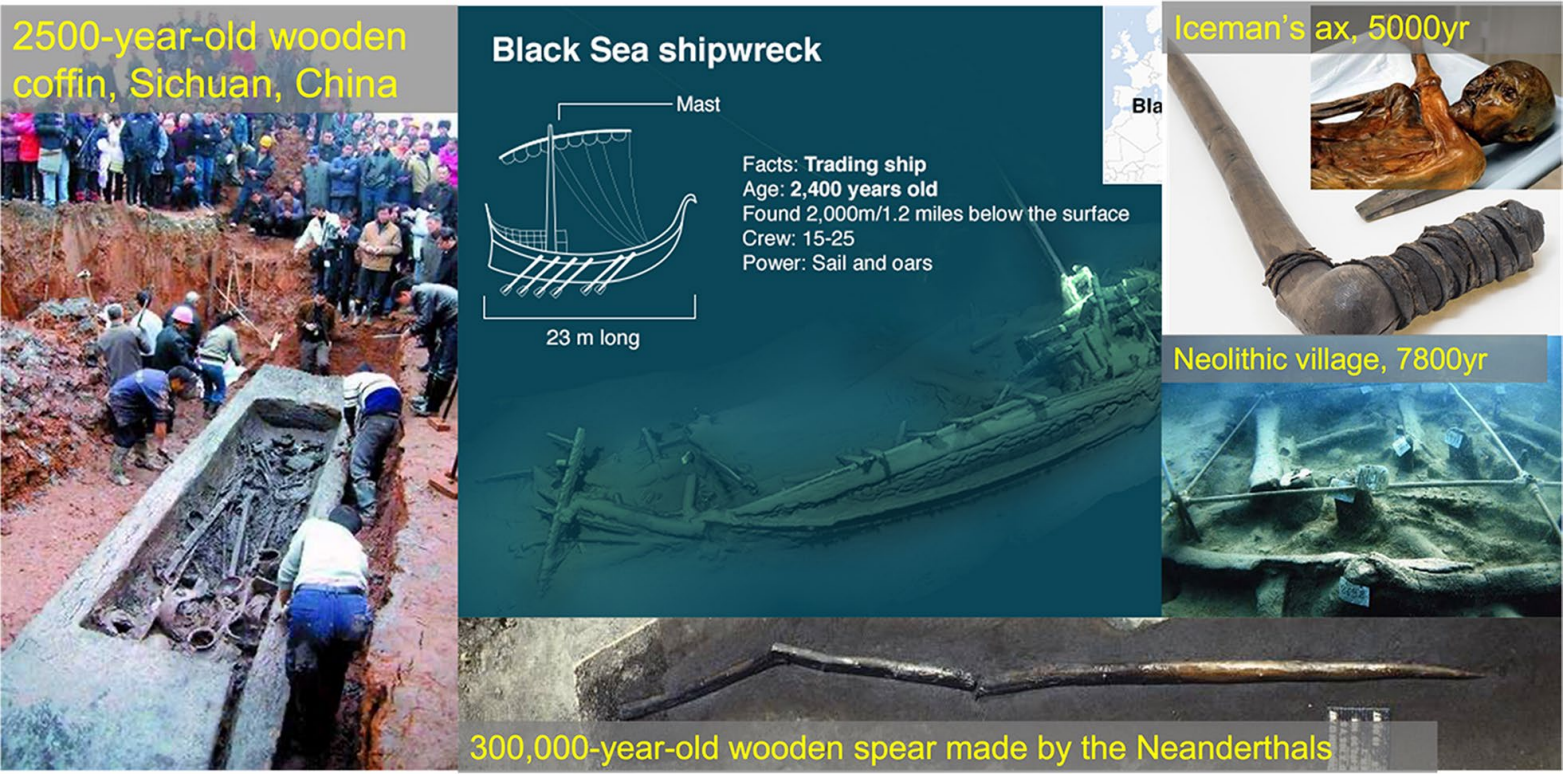
Ancient wood of human origin, thousands to hundreds of thousands of years. Photo credits: china.org.cn, BBC, Museo Archeologico dell'Alto Adige, Maria Antonietta Fugazzola Delpino, P. Pfarr

**Fig. 21:**
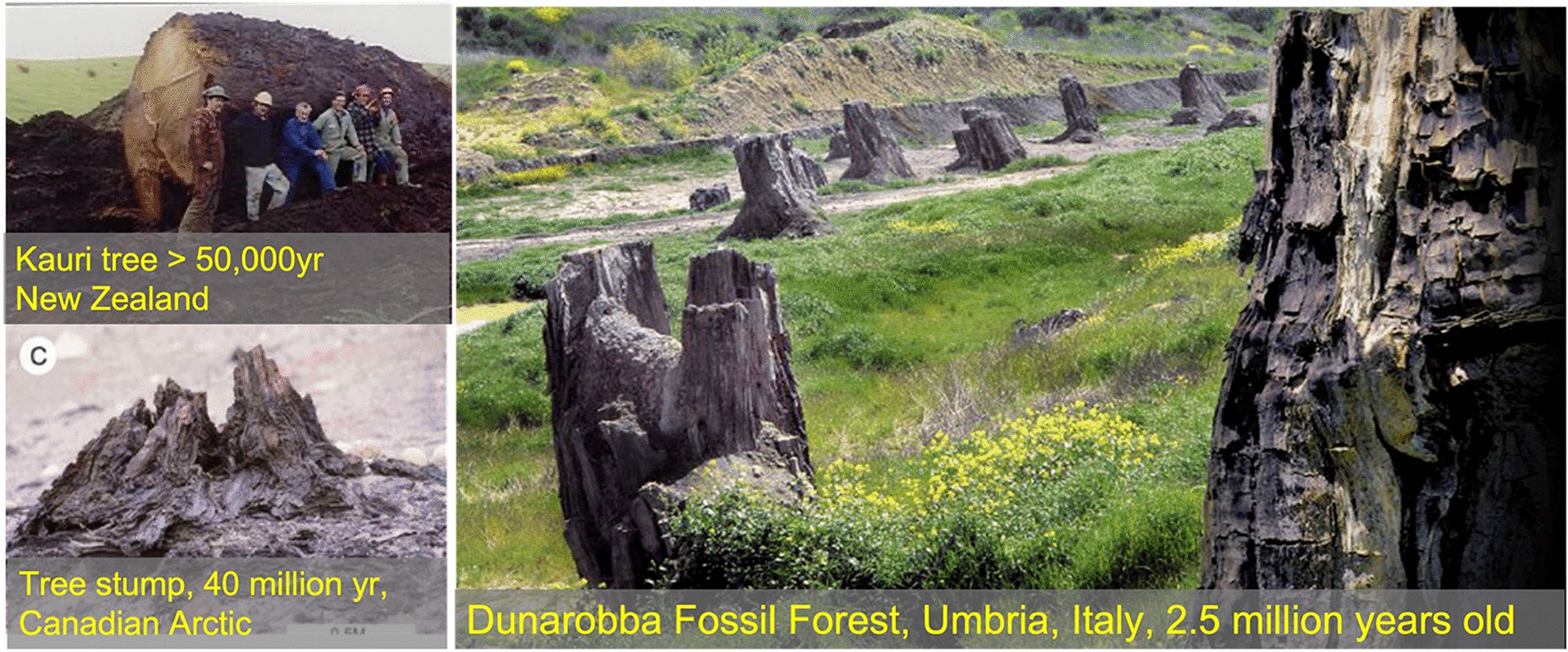
Ancient wood of geological origin. Photo credits: unknown, William Hagopian via G. Mustoe, umbriatourism.it

In our view, the most compelling evidence of the potential for long-term whole wood preservation comes from ‘natural’ experiments where ancient wood of archeological or geological origin, was found in good condition after hundreds, thousands, tens of thousands, hundreds of thousands, or even millions of years. A few examples are listed below (Figs. 19, 20, 21):

1. Wooden blocks in an Australian landfill after 46 years of burial [15]. (Low oxygen)
2. 100-year or older logs found at the bottom in many rivers/lakes around the world. (Anaerobic due to water logging)
3. Stockpiles of wood in the semi-arid region of America West. (Dry)
4. 800-year-old Wooden poles in Anasazi (Ancestral Puebloan) structure, embedded in stone-mud adobe, Cliff Palace, Mesa Verde National Park, Colorado. (Dry)
5. 2500-year-old wooden coffin from the Warring States Period (475–221 BC) found 1.5 m below the surface in red clay in Sichuan, China. (Anaerobic due to clay sealing)
6. 2400-year-old Greek Merchant ship sunken at the bottom of the Black Sea. (Low-oxygen bottom water)
7. 5000-year-old human body, Ötzi the iceman, together with his wooden tools and undigested food in his stomach, found in an Alpine glacier, Italy-Austria Border. (Cold)
8. 7800-year-old Neolithic settlement submerged under Lake Bracciano, Lazio, Italy. (Anaerobic due to waterlogging and silt)
9. The 300,000-year-old wooden spears used by the Neanderthals, Schöningen, Germany. (peat/lignite)
10. Ancient Kauri trees older than 50,000 years (beyond carbon dating) excavated from wetland 1–2 m below surface in New Zealand. (Anaerobic)
11. 2.5 million-years-old wood stumps found in a clay quarry in Umbria, Central Italy (anaerobic due to clay sealing)
12. 40 million years old wood stumps freely standing on a beach at Axel Heiberg Island, Canadian Arctic. (Currently cold, likely preceded by past waterlogging condition during warm periods)

### Pathways to semi-permanent wood preservation

**Fig. 22:**
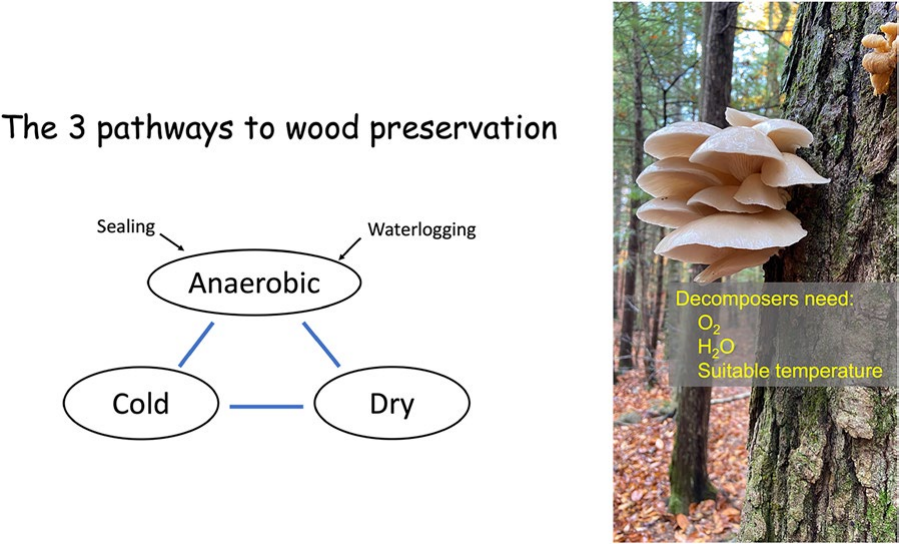
Three major pathways to prevent wood decomposition: (1) Anaerobic condition that can be created by sealing with low permeability material such as clay or waterlogging; (2) Cold; (3) Dry. Any one of these 3 conditions, if ensured in high fidelity, will be sufficient to preserve wood. This is fundamentally because the main wood decomposers such as fungi and insects need all three ingredients to survive and thrive: oxygen, suitable temperature, and moisture. The 7 versions of Wood Vaults are designed to realize one or more of these 3 pathways. Photo credit: Susan Pike

The examples listed above are mostly associated with clay-enclosed or water-logged anaerobic burial environment and the rest with cold or dry conditions. This is consistent with the basic biology of decomposition because the main decomposers such as fungi and insects need all of the following environmental conditions to survive and thrive: (1) oxygen, (2) suitable temperature, (3) moisture. This piece of fundamental biology leads to 3 major pathways to prevent wood decomposition (Fig. 22): (1) Anaerobic, (2) Cold, (3) Dry.

Any one of these 3 pathways, if ensured in high fidelity, will be sufficient to preserve wood semi-permanently. The goal of Wood Vault design is to realize one or more of these 3 pathways. Thus, our 7 versions of Wood Vaults can be classified based on these 3 environmental factors:

1. *Anaerobic* Version 1 (burial mounds), Version 2 (Underground), Version 3 (Super Vault), Version 4 (Shelter, if sealed), Version 5 (AquaOpen and AquaVault), Version 6.2 (DesertVault),
2. *Dry* Version 4 (Shelter, if non-sealed/ventilated), Version 6, Version 7 FreezeVault (freezing condition is simultaneously dry in absolute humidity)
3. *Cold* Version 7 FreezeVault

We note that the best waterlogging conditions are generally accompanied by silty environments such as bogs, wetland and peatland. In contrast, environments with free water movement such as at the bottom of the Black Sea, the Baltic Sea and the Great Lakes is likely to have low enough oxygen levels to enables wood preservation to some degree, but may or may not be anaerobic enough to ensure the long-term preservation needed for climate purpose. The relatively anaerobic bottom water of the Black Sea is caused by vertical stratification, but free-flowing water will inevitably have some amount of mixing, no matter how small it is. An exception is where the sedimentation rate is high so the wood logs can be buried before decay, as illustrated in Wood Vault Version 5.1 (AquaOpen, Fig. 10). Our AquaVault design calls for an artificial wrapping which may be able to circumvent the limitation of AquaOpen. Another caution is the lack of reinforcibility in the future discussed above. Thus, more caution is needed before large-scale implementation in aquatic environments. With our current understanding, terrestrial or wet + silty stagnant environments are more reliable at ensuring anaerobic condition.

In summary, for a Wood Vault enclosed in low-permeability soil, the anaerobic, stagnant sub-terranean environment ensures the durability of buried wood by minimizing biological, physical and chemical activities, ultimately leading to a preservation state of quasi-geological nature. We emphasize the critical importance of air-tight sealing, which leads to ultimate anaerobic condition. The initial oxygen at fresh burial is not a problem, because it would take the decomposition of only a tiny amount of organic matter to consume it.

Research with lab experiments and real-world projects will be needed to establish the best practice and define what is ‘sufficiently-good’ burial condition that balances cost and preservation under various soil and hydrological settings. In this vein, we emphasize the paramount importance of the practicality of any proposed solution, including low-cost, wide availability of the material such as clay/mud, ease of operation, suitability for local people and stakeholders, and environmental and social impact.

## Implication for the future: a thermostat to manage the climate system?

**Fig. 23:**
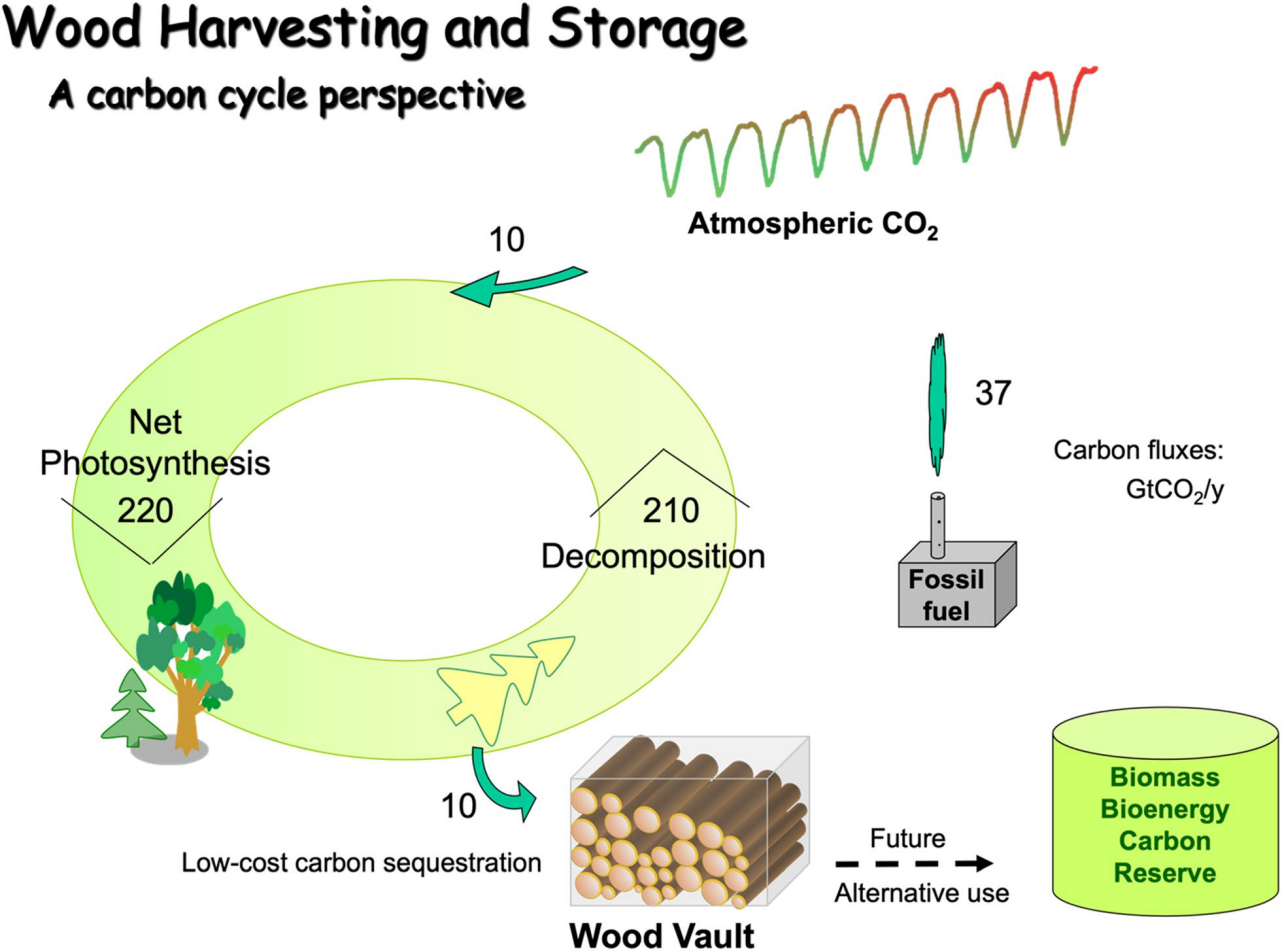
Wood Harvesting and Storage (WHS) siphons off a sustainable fraction of the biosphere production in the form of harvested wood and stores it in engineered Wood Vaults to prevent decomposition, forming an effective carbon sink. WHS is a ‘near-perfect’ reversal of fossil fuel emissions by accelerating the slow natural biomass burial process of fossil fuel formation. The stored woody biomass is not only a carbon sink to mitigate current climate change, but also a valuable resource for the future that can be used as biomass, bioenergy, and carbon supply. A WHS carbon sequestration rate of 10 GtCO_2_ y^−1^ is less than 5% of terrestrial net primary productivity (NPP: net photosynthesis)

On longer time horizons, the stored wood is also a reserve of biomass, bioenergy and carbon, should future needs arise (Fig. 23). For example, intentionally preserved wood logs in mill ponds or lakes, or naturally buried logs in riverbeds and muddy soil are a priced raw material for making high quality furniture. In another example, the current bioenergy/wood pellet industry is hampered by issues of reliability and sustainability of wood supply, and WHS stored wood would provide a buffer, i.e., more predictable wood source on which a sustainable bioenergy industry can be based. A main practical constraint is of course the cost of this additional buffer step.

**Fig. 24:**
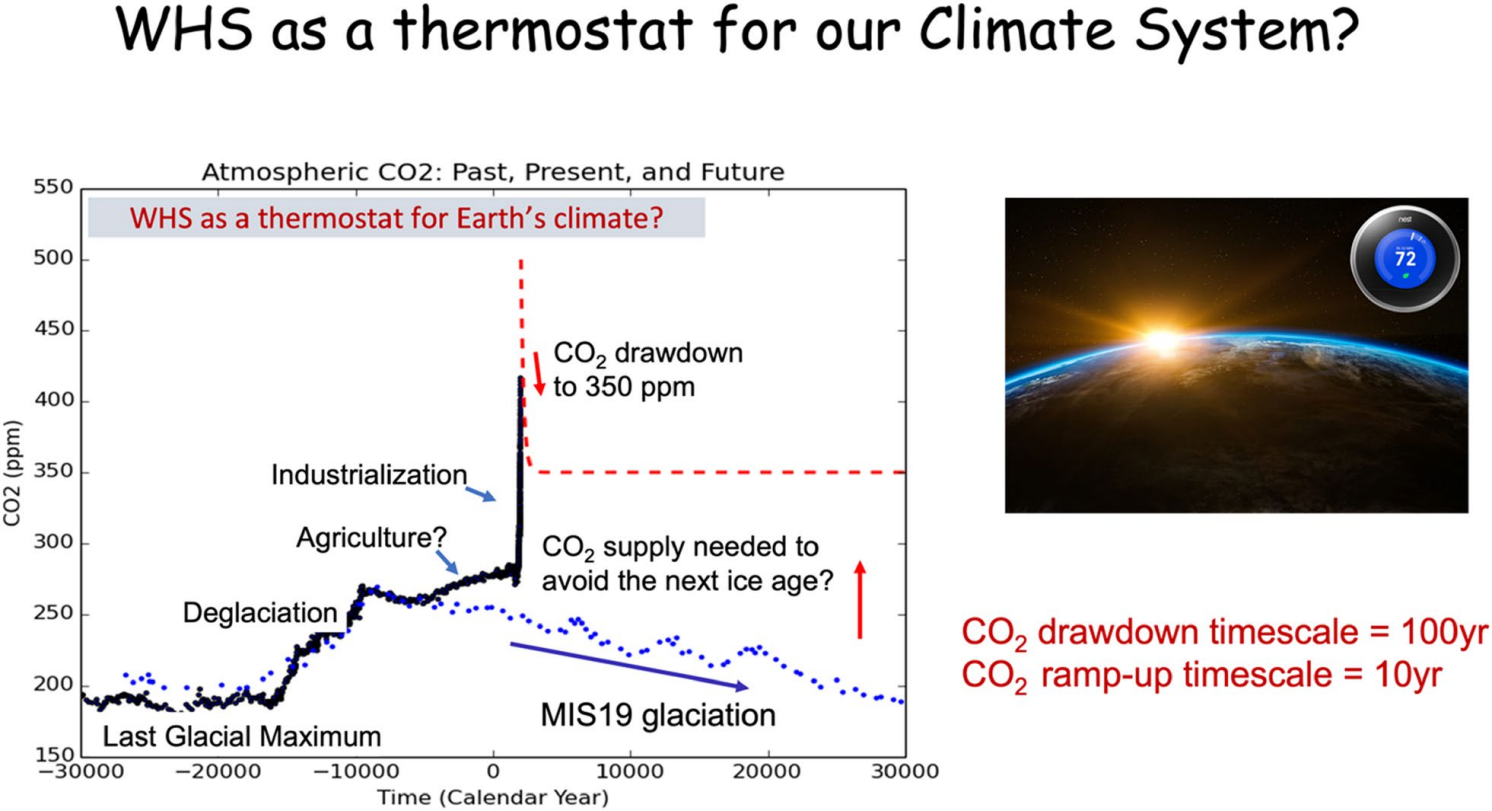
WHS as a thermostat for managing the Earth’s climate. Sustainable Wood Harvesting and Storage helps to remove CO_2_ and locks it away semi-permanently as a biomass/bioenergy/carbon reserve, which can be used as a CO_2_ supply should astronomical forcings drive the climate into an ice age in the future. CO_2_ data from Antarctica ice cores [29] (800,000 years ago to 1999) and Mauna Loa Observatory (1959–2021) [30] plotted in black dots, while red dots indicate a future scenario of an exponential decrease from 500 to 350 ppm [31], starting from 2050 with an e-folding timescale of 200 years. Blue dots are CO_2_ during the Marine Isotope Stage 19 (MIS19), shifted by 777,000 years as an analog of orbitally driven climate indicator, known as the Milankovitch Theory [22]. Identified periods in the CO_2_ data include the Last Glacial Maximum (LGM), the penultimate deglaciation, a Holocene CO_2_ rise of 20 ppm over the last 8000 years that has been hypothesized as consequence of agriculture (the Early Anthropogenic Hypothesis [25, 26]), and industrialization

On even longer time horizon of thousands of years or longer, after current climate warming trend gets under control as the society transitions successfully to renewable energy, should the natural rhythm of ice-age cycles kick the Earth’s climate into a glaciation period [22] (Fig. 24), that is, the opposite problem of current global warming, the buried wood can be taken out, used for energy while releasing CO_2_ to keep the Earth’s climate from diving into an ice age, particularly effective in light of the dominant role of CO_2_-climate feedback on ice age cycles [23–25].

This leads to a philosophical comment from the perspective of Earth system history. The current climate crisis has been caused by fossil fuel burning since the Industrial Revolution, on top of the disturbance to the global carbon cycle by agriculture that may have already delayed the inception of the next ice age [26]. These and other human activities are leading to a distinct geological epoch named the Anthropocene [27].

Wood Vault stores wood for carbon sequestration for now, while providing a biomass/bioenergy/carbon reserve for the future. The carbon reserve functionality alone allows WHS to be a useful management tool to adjust Earth’s temperature, acting as a thermostat. The extent and speed at which this thermostat can work is not totally clear, but should be significant given the basic fact that the Net Primary Productivity (NPP) of the terrestrial biosphere is 220 GtCO_2_ y^−1^ (60 GtC y^−1^), of which woody biomass NPP is 70 GtCO_2_ y^−1^ (one third of total) [3], and a sustainable harvesting potential for WHS up to 10 GtCO_2_ y^−1^ [4] (less than 5% of NPP), compared to current fossil fuel emission rate of 37 GtC y^−1^. The 10 GtC y^−1^ ‘practical’ potential has taken into account current land use and conservation needs. The time scale of CO_2_ “drawdown time” is dictated by sustainable wood harvest rate and is on the order of 100 years (1000 GtCO_2_ divided by 10 GtCO_2_ y^−1^, assuming the target is to remove 1000 GtCO_2_, or 130 ppm CO_2_ concentration, approximately fossil fuel carbon accumulated in the atmosphere since Industrialization, excluding the carbon sinks [28]).

This CO_2_ “drawdown time” is rather quick on geological time scale, suitable for removing CO_2_ for the current climate crisis. In the opposite direction when the stored wood is used to increase atmospheric CO_2_, the “ramp-up time” is probably on decadal time scale as we can burn the stored wood quickly if needed. Our discussion here only concerns a ‘thermostat’ for CO_2_, not temperature which would involve additional slow processes such as glacial melting and rebuilding, with very long timescales, but inclusion of these factors is beyond the scope of this paper.

## Conclusions

Wood Vault can be an efficient tool to lock down and sequester carbon reliably, using a variety of wood sources. Many sizes and versions of Wood Vault are possible. Most pieces of the technology already exist, but they need to be put together efficiently in practice. Some uncertainties need to be addressed, including how the durability and permanence of buried wood depends on detailed burial methods and burial environment, but the science and technology are known well enough to believe the practicality of the method. The high durability, verifiability and relatively low-cost makes it an attractive option in the current global carbon market.

Because WHS relies on trees to capture CO_2_ from the atmosphere, but the rate of photosynthesis and land availability are limited, there is an opportunity loss if wood storage is not carried out as soon as the wood is available because it would otherwise decompose into CO_2_. On the ground, this loss of opportunity manifests itself as waste wood rots in a landfill, hurricane damaged trees are collected and burned in a Florida neighborhood, trees are burned in a California forest, and so on. This leads to a SENSE of URGENCY. Assuming we can sustainably sequester 10 GtCO_2_ y^−1^, but we delay action for 10 years, we would have lost the opportunity to sequester 100 GtCO_2_.

Woody biomass stored in Wood Vaults is not only a carbon sink to combat the current climate crisis, but also a valuable resource for the future that can be used as biomass/bioenergy. The quantity of this wood utilization can be controlled carefully to maintain a desired amount of CO_2_ in the atmosphere to keep the Earth’s climate from diving into the next ice age. The CO_2_ drawdown time is on the order of 100 years while the ramp-up time of this WHS thermostat is a decade.

In conclusion, WHS provides a powerful tool for managing our Earth system, which will likely remain forever in the Anthropocene.

## Data Availability

No new data was produced in this work. All derived data are included in the tables and text.

## Appendix

### Wood availability: regional to global estimates

We consider two types of wood sources: (1) opportunistic sources; (2) harvest from sustainably managed forests. For opportunistic wood, Perlack et al. (2005) analyzed data from US Forest Inventory Analysis (FIA) in the context of biomass for energy use. We extracted information on unexploited wood sources from the current forestry wood use, summarized in Table

**Table 5:** Unexploited wood sources in the US (Perlack et al. [6]; dry ton converted to wet tonne or tCO_2_ using a conversion factor of two)

Sourc**e**		Availability per area tCO_2_ ha^−1^	State MD (25,000 km^2^) MtCO_2_ y^−1^	US (unexploited/total) MtCO_2_ y^−1^	Permanence and durability after WHS storage	Current use
Urban wood residue (56 out of 124Mt total)	Woody yard trimmings (natural wood) MSW	0.2	0.5	4/20 (4 out of total of 20)	1000+ years	Partially used for mulch, energy
	Construction residue			17/23	100+ years	
	Demolition debris			23/55		
	Wood MSW (Furnitures +)			12/26	100+ years	
Forest thinning (128 Mt)	Thinning for fire prevention (fuel treatment)	0.4	1	120	1000+ years (large pieces) 100 years (smaller pieces)	Not common (needs policy and investment)
Logging residue		0.3	0.75	93	100+ years	Partially used for mulch, energy
Other removals (land clearing precommercial treatment)		0.12	0.3	35		
Forest products industry wastes (Mill residues)		0.05	0.125	16/321		
Total		1.1	2.8	328		

Available = Generated-(Recovered + Combusted + unusable). Unusable sources such as contaminated wood were already excluded. Potential availability per unit area is assumed to be distributed over US forested land of 3 Mkm^2^

^5^. Not included are other possibilities such as damaged wood from storm-blowdown and insect outbreaks for which there are no comprehensive estimates.

The total US unexploited wood in green megatonnes is: 56 (urban) + 35 (other removal) + 128 (thinning) + 93 (logging) + 16 (mill) = 328 Mt y^−1^. If we distribute this over the US forested area of approximately 3 Mkm^2^, the wood availability is: 0.2 (urban) + 0.12 (other removal) + 0.43 (thinning) + 0.3 (logging) + 0.05 (mill) = 1.1 t ha^−1^. This leads to potential availability per unit area of 328/300 = 1.1 tCO_2_ ha^−1^.

The unexploited urban and other removal are immediately available, which add to 0.32 t ha^−1^. Because carbon value will likely divert some more wood from some other current uses such as making mulch which currently utilizes most urban tree removal (tree yard trimmings: 16 of 20 Mt), we assume a 0.4 tCO_2_ ha^−1^ to be practically available immediately.

It is interesting to compare these bottom-up estimates with the top-down estimates based on forest productivity of Zeng et al. [4]. In the high 11 GtCO_2_ y^−1^ (3 GtC y^−1^) global top-down scenario, Zeng et al. [4] found that the US can contribute 500 MtCO_2_ y^−1^ (0.14 GtC y^−1^) for the US, slightly higher than the inventory-based estimate above. Distributed over 3 Mkm^2^ of forest land, this leads to an areal harvesting intensity of 1.7 tCO_2_ ha^−1^. In their low 3.7 GtCO_2_ y^−1^ (1 GtC y^−1^) global scenario, the US can contribute^−1^170 MtCO_2_ y^−1^at a harvesting intensity of 0.6 tCO_2_ ha^−1^ over its 3 Mkm^2^ forested land.

Globally, for their lower 3.7 GtCO_2_ y^−1^scenario, Zeng et al. [4] stated that 4.4 tCO_2_ ha^−1^ (1.2 tC ha^−1^) on 8 Mkm^2^ leads to 1 GtC y^−1^. The feasibility of such wood collection rate can be viewed from forest productivity. The 4 tCO_2_ ha^−1^ rate is about 10% of the NPP of a typical temperate forest [4]. Collecting wood at the 4 tCO_2_ ha^−1^ on an area of 9 million km^2^ (about the area of the US) would sequester 3.7 GtCO_2_ y^−1^ (1 GtC y^−1^, 1 wedge of of Pacala and Socolow [32], or 10% of global total fossil fuel emissions in 2020). The urban wood residual availability of 0.4 tCO_2_ ha^−1^ is only 1% of the NPP of a typical temperate forest. The sustainability of these relatively low intensity harvest is thus likely to be highly feasible, though caution is still needed in overall resource planning, especially from government and regulatory agency perspective.

## Footnotes

1 A tonne of wet biomass, also known as a green tonne, contains about 0.5 dry tonne biomass. Dry biomass contains about 50% carbon (C), with the rest mostly oxygen (O) and hydrogen (H). Thus, material in 1 green tonne contains about 25% carbon which is oxidized to about 1 tCO_2_ (tonne of CO_2_). 1 tC = 3.67 tCO_2_ (approximately 4 tCO_2_) as the molecular weight ratio of CO_2_:C = 44:12. We also approximate 1 short ton in US literature with 1 metric tonne for estimates in this paper. In summary, 1 wet/green tonne of wood has approximately a volume of 1 cubic meter while sequestering 1 tCO_2_. Such approximation lends simplicity in our high-level assessment and is within the uncertainty range of natural variations in tree species, wood moisture content, and other factors.
